# Comparison of randomized controlled trials discontinued or revised for poor recruitment and completed trials with the same research question: a matched qualitative study

**DOI:** 10.1186/s13063-019-3957-4

**Published:** 2019-12-30

**Authors:** Matthias Briel, Benjamin Speich, Erik von Elm, Viktoria Gloy

**Affiliations:** 1 Basel Institute for Clinical Epidemiology and Biostatistics, Department of Clinical Research, University of Basel and University Hospital Basel, Basel, Switzerland; 2 Department of Health Research Methods, Evidence, and Impact, McMaster University, Hamilton, ON Canada; 3 Cochrane Switzerland, Center for Primary Care and Public Health (Unisanté), University of Lausanne, Lausanne, Switzerland

**Keywords:** randomized controlled trials, early termination of clinical trials, poor recruitment, systematic reviews, qualitative analysis

## Abstract

**Background:**

More than a quarter of randomized controlled trials (RCTs) are prematurely discontinued, mostly due to poor recruitment of patients. In this study, we systematically compared RCTs discontinued or revised for poor recruitment and completed RCTs with the same underlying research question to better understand the causes of poor recruitment, particularly related to methodological aspects and context-specific study settings.

**Methods:**

We compared RCTs that were discontinued or revised for poor recruitment to RCTs that were completed as planned, matching in terms of population and intervention. Based on an existing sample of RCTs discontinued or revised due to poor recruitment, we identified matching RCTs through a literature search for systematic reviews that cited the discontinued or revised RCT and matching completed RCTs without poor recruitment. Based on extracted data, we explored differences in the design, conduct, and study settings between RCTs with and without poor recruitment, separately for each research question using semi-structured discussions.

**Results:**

We identified 15 separate research questions with a total of 29 RCTs discontinued or revised for poor recruitment and 48 RCTs completed as planned. Prominent research areas in the sample were cancer and acute care. The mean number of RCTs with poor recruitment per research question was 1.9 ranging from 1 to 4 suggesting clusters of research questions or settings prone to recruitment problems. The reporting quality of the recruitment process in RCT publications was generally low. We found that RCTs with poor recruitment often had narrower eligibility criteria, were investigator- rather than industry-sponsored, were associated with a higher burden for patients and recruiters, sometimes used outdated control interventions, and were often launched later in time than RCTs without poor recruitment compromising uncertainty about tested interventions through emerging evidence. Whether a multi- or single-center setting was advantageous for patient recruitment seemed to depend on the research context.

**Conclusions:**

Our study confirmed previously identified causes for poor recruitment, i.e., narrow eligibility criteria, investigator sponsorship, and a reduced motivation of patients and recruiters. Newly identified aspects were that researchers need to be aware of all other RCTs on a research question so that compromising effects on the recruitment can be minimized and that a larger number of centers is not always advantageous.

## Background

Evidence-based health care relies on high-quality clinical research. Randomized controlled trials (RCTs) are the method of choice to assess preventive and therapeutic interventions and are a cornerstone in the final phase of drug development and in comparative effectiveness research. Conducting high-quality RCTs, however, is challenging. More than a quarter of RCTs do not reach the planned sample size, mostly due to poor recruitment of patients, and are prematurely discontinued [1, 2]. Investigator-initiated RCTs are particularly prone [1]. Implications of poor patient recruitment and premature discontinuation of RCTs are that up to 70% of such trials remain unpublished, root causes of recruitment difficulties are not shared with the scientific community and may therefore be repeated in the future, research questions remain unanswered, and substantial amounts of scarce research resources are wasted [1, 3].

To better understand the causes of poor recruitment, previous quantitative approaches using RCT protocols and registry information [1, 2] as well as qualitative analyses from published reports and semi-structured interviews with trialists and other stakeholders in clinical research have already provided important insights [3, 4]. That is, for instance, that investigator-initiated RCTs or RCTs in the acute care setting were found to be at much higher risk for discontinuation due to poor recruitment than industry-sponsored RCTs or RCTs in non-acute care settings [1, 2]; or that insufficient preparation, overly narrow eligibility criteria, and prejudiced views of recruiters and patients on trial interventions are common reasons for poor recruitment [3]. As suggested by others previously [5], we undertook a systematic comparison of RCTs discontinued or revised for poor recruitment and RCTs completed as planned with the same underlying research question. We aimed to provide additional evidence on potential causes for poor recruitment specifically related to design aspects and context-specific study settings of RCTs.

## Methods

This is a matched qualitative study comparing RCTs that did not reach 90% of their originally planned sample size due to poor recruitment (cases; discontinued or revised RCTs with poor recruitment) to RCTs completed as planned (controls without poor recruitment) matching in terms of patient population and experimental intervention. The study is reported according to the Standards for Reporting Qualitative Research (SRQR, http://www.equator-network.org/reporting-guidelines/srqr/) as described in Additional file 1.

### Identification of discontinued and matching completed RCTs

In a previous study, we included 20 RCTs that were discontinued or revised for poor recruitment with the planned sample size being reported in a publication [1]. For these studies, we aimed to find matching RCTs reaching at least 90% of their originally planned sample size. We searched for systematic reviews that cited the discontinued or revised RCT using the “times cited” function in Web of Science (times cited “view all of the articles that cite this one” https://apps.webofknowledge.com) in January 2016. One reviewer (VG) screened the titles, abstracts, and full texts of potentially eligible systematic reviews for relevance. An eligible systematic review had to describe a literature search conducted in at least one electronic database (e.g., Medline), include RCTs, and had to have a research question similar to that of the discontinued or revised RCT. If more than one systematic review were eligible, we chose the most up-to-date, comprehensive systematic review (frequently a Cochrane review). If no systematic review was identified, we searched by the same means for similar narrative reviews. In case VG was in doubt about the eligibility of a systematic review, she involved a second reviewer (MB) for discussion and consensus decision.

From each eligible systematic review we retrieved the full text articles of included RCTs (i.e., potentially eligible matching RCTs) and collected a small set of preliminary data. These included whether the RCT was discontinued or revised for poor recruitment, planned and actually achieved sample size, the patient population, and the experimental and control intervention. This was done by two methodologically trained investigators (VG, BS) working independently and in duplicate. Any disagreements were resolved by consensus and, if needed, through involving another investigator (MB). The matching criteria (inclusion criteria) were the tested intervention and included patient population, which needed to be similar enough so that the RCTs could be included in the same meta-analysis. Other trial characteristics such as comparator interventions, outcomes of interest, and trial settings already qualified as factors potentially associated with poor recruitment.

### Data collection

The following data were collected from the eventually included RCTs: study design (e.g., superiority or non-inferiority trial, factorial or parallel design, allocation ratio), sample size calculation, allocation concealment, blinding, reporting quality of the recruitment process, eligibility criteria, trial sponsor, country and place of patient recruitment, recruitment period, reporting of recruitment networks, support from a clinical trial unit or contract research organization, study population, interventions, comparators, and primary outcome. In addition, we also extracted self-reported reasons (if any) for poor recruitment of the discontinued or revised RCTs. Data were collected by one investigator (VG or BS) and checked by another (VG, BS or MB). Any disagreements were resolved by consensus.

### Data analysis

We explored differences in the design, conduct. and study settings between RCTs with and without poor recruitment, separately for each research question, thus context-specific, using semi-structured discussions (MB, BS, and VG). In a first round, we gathered differences between RCTs with and without poor recruitment, based on our extracted data; we systematically went through a pre-specified checklist of items potentially associated with poor recruitment, including eligibility criteria, trial sponsor, single versus multiple centers, etc. (Additional file 2); and discussed each item in turn followed by observations beyond the checklist items (e.g., the chronology of RCTs building up the evidence base for a specific research question). When we reached agreement among us, the identified differences and potentially relevant observations were captured by VG in free-text form. In a second round, we reflected on the identified differences between RCTs with and without poor recruitment and other relevant observations for each research question and integrated the relevant information in order to come up with an explanation why for a certain research question one or more RCTs had serious recruitment problems while others did not have such problems. In addition, we considered the self-reported reasons for poor recruitment (if any).

### Researchers’ reflexivity

The three researchers who carried out the analysis have diverse disciplinary background and training such as medicine/clinical epidemiology (MB), nutrition/health technology assessment (VG), and epidemiology/public health (BS). To better understand specific characteristics of oncology trials, we involved a practicing oncologist in the respective discussions. For the other topics, the analysis team members felt that they had sufficient knowledge to assess trial characteristics, and their methodological expertise with clinical trials likely strengthened the analysis. None of us knew any of the included RCTs or their investigators. During analysis, all researchers worked together as a team and extensively discussed the data interpretation to minimize bias.

## Results

### Study sample

**Fig. 1:**
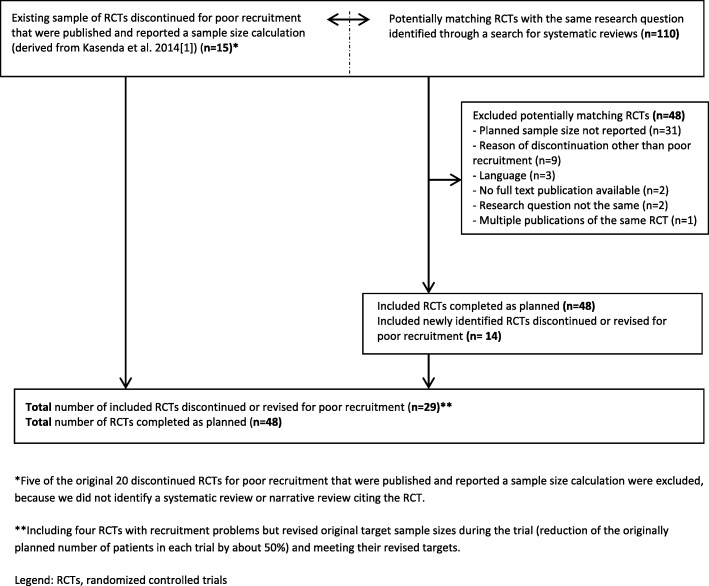
Selection of included randomized controlled trials. *Five of the original 20 discontinued RCTs for poor recruitment that were published and reported a sample size calculation were excluded, because we did not identify a systematic review or narrative review citing the RCT. **Including four RCTs with recruitment problems but revised original target sample sizes during the trial (reduction of the originally planned number of patients in each trial by about 50%) and meeting their revised targets. *RCT* randomized controlled trial

In a previous study, there were 20 published RCTs discontinued or revised for poor recruitment that reported the planned sample size [1]. For five of these RCTs our literature search could not identify a systematic or narrative review that cited the discontinued or revised RCT (Fig. 1). For the remaining 15 RCTs discontinued or revised due to poor recruitment representing 15 different research questions, we identified 110 matching RCTs. We excluded 48 RCTs because the planned sample size was not reported (*n* = 31); the RCT was discontinued for another reason than poor recruitment (*n* = 9); the article was not published in English (*n* = 3); the research question was not similar enough (*n* = 2); there was no full-text publication available (*n* = 2); or the same results were published multiple times (*n* = 1). Of the 62 matching RCTs, 48 were completed as planned and 14 were newly identified RCTs discontinued or revised for poor recruitment. Hence, for 15 separate research questions a total of 77 RCTs (29 with and 48 without poor recruitment) were available for comparative analyses. Twenty-five of the 29 RCTs with poor recruitment explicitly reported that they were discontinued due to recruitment problems; the remaining four RCTs [6–9] had recruitment problems but revised their original target sample sizes during the trial (reduction of the originally planned number of patients in each trial by about 50%) and met the revised targets. References of all included RCTs are provided in Additional file 3.

### Research questions, recruitment, and reporting quality

Medical areas of the 15 included research questions were cancer research (*n* = 5), research in acute care (*n* = 8; including preterm infants [*n* = 2] and laboring women [*n* = 1]), surgery (*n* = 1), and infectious diseases (*n* = 1). The mean number of RCTs with poor recruitment was 1.9 per research question ranging from 1 to 4. Table 1 summarizes the recruitment characteristics for the RCTs with and without poor recruitment across all research questions. In general, RCTs with poor recruitment recruited fewer patients per year per recruiting center compared to RCTs that were completed as planned. More detailed recruitment characteristics for each research question and for all included RCTs, as well as general study characteristics are provided in Additional files 4, 5 and 6.

**Table 1:** Recruitment characteristics of included randomized controlled trials with and without poor recruitment across research questions

	RCTs with poor recruitment, *n* = 29	RCTs without poor recruitment, *n* = 48
Originally planned number of patients (median; IQR)	395 (72–600)	272 (22–500)
Number of patients randomized (median; IQR)	150 (41–329)	306 (23–559)
Duration of recruitment period in months (median; IQR)	39 (10–47)	24 (7–45)
Number of patients recruited per year and center^a^ (median; IQR)	3 (1–5)	7 (1–25)
Trial sponsor (industry n (%) / investigator n (%))	12 (41%) / 17 (59%)	20 (42%) / 27 (56%) / NR 1 (2%)

*IQR* interquartile range (25th and 75th percentile), *NR* not reported, *RCT* randomized controlled trial

^a^Rough estimate for recruitment speed based on own calculations (number of patients recruited divided by recruitment duration and number of study centers); not adjusted to the time a site was actually open for recruitment, because this was not reported in the publications of included trials)

The reporting of the recruitment process was generally in included RCT publications with little detail. None of the articles reported on who actually recruited patients or the anticipated prevalence of eligible patients. Only 6% (5/77) of the RCTs reported on the anticipated recruitment duration, 51% (39/77) reported the location where patients were recruited, 27% (21/77) provided a detailed patient flow, and 90% (69/77) reported the actual recruitment period or duration (Additional file 7).

### Comparison of RCTs with and without poor recruitment

Table 2 summarizes the differences observed between RCTs with and without poor recruitment for each research question as well as our context-specific conclusions on the possible reasons for poor recruitment. The most recurrent theme across research questions was that, in RCTs with poor recruitment, eligibility criteria were substantially narrower than in RCTs without poor recruitment (research questions #1, #2, #3, #4, #9, #12, #13 in Table 2).

**Table 2:** Differences between randomized controlled trials with and without poor recruitment and conclusions on the possible reasons for poor recruitment

Groups of RCTs with a common research question	Observed differences with respect to study characteristics (design, conduct, sponsor) between RCTs with and without poor recruitment	Context-specific conclusions on the possible reasons for poor recruitment
#1: Surgical treatment compared to proton-pump inhibitors in gastroesophageal reflux disease RCTs with poor recruitment *n* = 2 RCTs without poor recruitment *n* = 2	1) Study sponsor: RCTs with poor recruitment were investigator-sponsored whereas RCTs without poor recruitment were industry-sponsored	Absence of industry sponsoring could mean less professional trial organization and limited funding in general; narrower eligibility criteria may correlate with overestimation of recruitment rates and with slow recruitment; and a higher burden for patients during follow-up may have lowered the motivation of reflux patients to participate
	2) Eligibility criteria: more restrictive in the RCTs with poor recruitment with respect to disease duration and duration of prior proton-pump inhibitor usage	
	3) Patient burden during follow-up: patient assessment during follow-up more frequent in one of the RCTs with poor recruitment (Anvari et al. 2011) than in the RCTs without poor recruitment (e.g., assessment of quality of life every 3 months vs. every 12 months)	
#2: Anthracyclines with or without any taxanes compared to anthracyclines plus any anticancer treatment or compared to single-agent taxanes in metastatic breast cancer RCTs with poor recruitment *n* = 3 RCTs without poor recruitment *n* = 3	1) National/international: RCTs with poor recruitment were conducted in one country (national) whereas the RCTs without poor recruitment were international (1 not reported)	Absence of an international network of recruiting centers may be associated with limited recruitment capacities for patients with metastatic breast cancer; narrower eligibility criteria may correlate with overestimated recruitment rates and with slow recruitment; and the fact that evidence on the effectiveness of anthracycline combination therapy from previous RCTs was already available may have compromised the motivation of recruiters in the RCTs with poor recruitment
	2) Eligibility criteria: more restrictive in 2 of 3 RCTs with poor recruitment (Bonneterre et al. 2004 and Bontenbal et al. 2005) with respect to adjuvant therapy prior to enrolment, i.e., adjuvant therapy had to be stopped at least 12 months before	
	3) Publication chronology/available evidence: RCTs with poor recruitment were published (years: 2004, 2005, and 2010) after 2 of the 3 RCTs without poor recruitment (Biganzoli et al. 2002 and Jassem et al. 2001); the other RCT without poor recruitment was published in 2005 (Nabholtz et al. 2005)	
#3: First-line treatment with the aromatase inhibitor exemestane compared to anastrozole in postmenopausal women with advanced breast cancer RCTs with poor recruitment *n* = 1 RCTs without poor recruitment *n* = 1	Eligibility criteria: more restrictive in the RCT with poor recruitment with respect to metastases and disease stage (visceral metastases only vs. advanced stage or any metastases in the RCT without poor recruitment)	Narrower eligibility criteria for postmenopausal women with advanced breast cancer may correlate with overestimated recruitment rates and with slow recruitment
#4: Antiarrhythmic agents compared to placebo or background therapy with beta-blocker in ventricular arrhythmia RCTs with poor recruitment *n* = 1 RCTs without poor recruitment * n* = 2	1) Intervention tested: RCTs without poor recruitment tested a new intervention, i.e., celivarone (Kowey et al. 2011) or azimilide (Dorian et al. 2004) whereas the RCT with poor recruitment [8] tested available drugs (beta-blocker, amiodarone, and sotalol)	The fact that already available antiarrhythmic drugs and no new drugs were tested may have compromised the motivation of recruiters or patients; recruitment capacities might have been insufficient due to too few study centers; and narrower eligibility criteria for patients with ventricular arrhythmia may correlate with overestimated recruitment rates and with slow recruitment
	2) Number of study centers: 3 to 4 times lower in RCT with poor recruitment	
	3) Eligibility criteria: more restrictive in the RCT with poor recruitment [8] with respect to usage of antiarrhythmic agents prior to enrolment and the implantable cardioverter defibrillator device (only one specific type allowed)	
#5: Prophylactic antibiotics compared to placebo or usual care in acute necrotizing pancreatitis RCTs with poor recruitment *n* = 3 RCTs without poor recruitment *n* = 0	No differences observed because only RCTs with poor recruitment were identified	The fact that 3 RCTs were discontinued due to poor recruitment in this research area suggests that it seems generally problematic to recruit patients with acute necrotizing pancreatitis, which is associated with a high mortality rate (vulnerable patients)
#6: Late postnatal (> 7 days) corticosteroid treatment compared to placebo or usual care in preterm neonates RCTs with poor recruitment *n* = 3 RCTs without poor recruitment *n* = 7	1) Number of study centers: all RCTs without poor recruitment were single-center studies, whereas 2 (Kari et al. 1993 and Doyle et al. 2006) of the 3 RCTs with poor recruitment were multicenter studies	The fact that 3 RCTs recruited poorly in this research area suggests that it seems generally problematic to recruit preterm neonates (vulnerable patients). In a setting with vulnerable patients, a single-center RCT may prove advantageous due to more motivated and better prepared/trained recruiters and closer monitoring of recruitment). Particularly problematic appeared the choice of primary outcome in Doyle et al. 2006; to focus on a potential side effect of the intervention may have compromised the motivation of recruiters and parents to include children in such an RCT. Equipoise may be compromised for RCTs conducted later in a group of RCTs addressing the same research question (leading to lower motivation of recruiters and parents), because evidence on the effectiveness of an intervention from previous RCTs was already available
	2) Primary outcome: the primary outcome chosen in one RCT with poor recruitment (Doyle et al. 2006) focused on a potential side effect of the intervention (major neurosensory disability), whereas the other trials had an effectiveness outcome	
	3) Publication chronology/available evidence: a large RCT with poor recruitment (Doyle et al. 2006) was the last published RCT in this area of research whereas all other RCTs (discontinued or completed) were published earlier and already showed benefits in terms of better respiration of infants	
#7: Ventilatory gas with nitric oxide compared to ventilator gas without nitric oxide in preterm neonates RCTs with poor recruitment *n* = 2 RCTs without poor recruitment *n* = 5	1) Number of study centers: The number of study centers was consistently smaller among the RCTs without poor recruitment	In a setting with vulnerable patients (preterm neonates) a smaller number of study centers within 1 country may prove advantageous due to less complex trial organization and recruitment monitoring and potentially more motivated and better prepared/trained recruiters.
	2) National/international: whereas all RCTs with poor recruitment were international studies, all but one RCT without poor recruitment (Hascoet et al. 2005) had a national setting	
#8: Primary angioplasty compared to on-site thrombolytic therapy in acute myocardial infarction (within 12 h) RCTs with poor recruitment *n* = 3 RCTs without poor recruitment *n* = 3	Target sample size: the number of patients planned to be enrolled was consistently higher in RCTs with poor recruitment	The fact that 3 RCTs recruited poorly in this research area suggests that it seems generally problematic to recruit acute care patients. The comparison of angioplasty (rapid patient transfer to center necessary) to on-site thrombolytic therapy was logistically very challenging at the time. All RCTs with poor recruitment were meant to include more patients than the completed RCTs, consequently requiring more centers and often international collaboration, which additionally increased challenges with trial logistics
#9: Moxifloxacin compared to other antibiotics in patients with pneumonia RCTs with poor recruitment *n* = 2 RCTs without poor recruitment *n* = 4	1) Patient population: both RCTs with poor recruitment included patients with hospital-acquired pneumonia, at least in part mechanically ventilated (potentially sicker/more vulnerable patients), while RCTs without poor recruitment included patients with community-acquired pneumonia without mechanical ventilation	Recruitment of sicker/mechanically ventilated patients appeared more challenging (informed consent etc.); patients who acquired pneumonia in a hospital may have been less motivated to participate in an RCT conducted at that hospital, particularly if severely affected (mechanically ventilated) In addition, all 6 RCTs were designed as non-inferiority trials, i.e., the tested antibiotics were not hypothesized to have superior effects but may have been cheaper or easier to apply, so patients with hospital-acquired pneumonia could not expect a more efficacious treatment and might have therefore been less motivated to participate. For the same reasons (sicker patients, hospital-acquired infection, non-inferiority design) recruiters may have been less motivated, too. The eligibility criteria required the application of a complex scoring system (e.g., APACHE score) to determine patient eligibility, this may have increased the burden for recruiters and reduced their motivation
	2) Eligibility criteria: one of the RCTs with poor recruitment used the APACHE (Acute Physiology And Chronic Health Evaluation) score to define eligible patients (Höffken et al. the RCTs without poor recruitment did not	
#10: Temozolomide (chemotherapeutic agent) alone or in combination with radiotherapy compared to no chemotherapy (e.g., radiotherapy), non-temozolomide-based chemotherapy or temozolomide at different doses in glioma patients RCTs with poor recruitment *n* = 1 RCTs without poor recruitment *n* = 3		In this group of RCTs with glioma patients, all trials were published after the completed and published RCT conducted by Stupp et al. 2005. Subsequent to this publication, it was probably difficult to recruit patients in RCTs that did not include the intervention that was shown to be superior in Stupp et al. 2005 (combination of radiotherapy and chemotherapy). However, neither the RCT with poor recruitment (Malmström et al. 2012) nor another RCT without poor recruitment (Wick et al. 2012) included such a combination therapy, both RCTs were very similar with respect to patient population (elderly) and compared interventions (temozolomide vs. standard radiotherapy). The only other RCT without poor recruitment compared temozolomide to another chemotherapeutic treatment in patients not suitable for radiotherapy (Brada et al. 2010)
#11: Capecitabine-based chemotherapy compared to non-capecitabine chemotherapy in metastatic breast cancer RCTs with poor recruitment *n* = 4 RCTs without poor recruitment *n* = 5	Intervention tested: except for 1 RCT with poor recruitment (Bachelot et al. 2011) the RCTs with poor recruitment allocated patients to either an oral monotherapy with capecitabine or an intravenous application of a capecitabine-free chemotherapy either as monotherapy or as combination therapy. In contrast, in all but 1 RCT without poor recruitment (O’Shaughnessy et al. 2001), the route of administration was identical for both treatment arms (always included an intravenous application) and capecitabine was (with the same exception of O′Shaughnessy et al. 2001) given as combination therapy. The RCT without poor recruitment O′Shaughnessy et al. 2001, similar to the RCTs with poor recruitment also allocated patients either to intravenous or oral treatment, but at the time of trial conduct, oral monotherapy with capecitabine was relatively new and a 2:1 randomization was done to make it more likely for patients to get allocated to the anticipated patients’ preferred treatment option	Patients’ preferences regarding the form of application might have played a role in this research area, i.e., patient recruitment in RCTs comparing different interventions that, in addition, use different routes of application is hampered if patients have preferences about the route of application and therefore are less willing to be randomized. Two other reasons for poor recruitment related to patients’ or recruiter preferences are possible, but could be specific for the respective RCT. (1) In 1 RCT with poor recruitment (Talbot et al. 2002) the tested drug was applied continuously whereas intermittently might have been preferred by patients. (2) In another RCT with poor recruitment (Stockler et al. 2011) the control treatment (cyclophosphamide, methotrexate, and fluorouracil, CMF) was, at the time, outdated and therefore the motivation of recruiters and patients might have been reduced. In the discussion authors stated: “The main limitations of our trial are the ambiguity of broad, pragmatic eligibility criteria and the use of an unfashionable control regimen.”(Stockler et al. 2011)
#12: Primary thromboprophylaxis (heparin) compared to placebo or usual care in ambulatory cancer patients receiving chemotherapy RCTs with poor recruitment *n* = 2 RCTs without poor recruitment *n* = 2	1) Study sponsor: both RCTs with poor recruitment were investigator initiated whereas both RCTs without poor recruitment were industry-sponsored	Testing thromboprophylaxis with heparin in cancer patients may be challenging in general, because the intervention was not focused on combating the cancer, and therefore recruiters’ and patients’ interest in such an RCT may be reduced and seen as burden; competing RCTs testing antitumor agents might be preferred. In addition, the absence of industry sponsoring could mean less professional trial organization and limited funding in general, and a focus on only 1 type of cancer (narrow eligibility criteria, Perry et al. 2002) may have contributed to poor recruitment
	2) Eligibility criteria: only glioma patients were eligible in 1 of the RCTs with poor recruitment (Perry et al. 2002) while both RCTs without poor recruitment included patients with various types of cancer	
#13: Recombinant tissue plasminogen activator (rtPA) compared to placebo in acute ischemic stroke RCTs with poor recruitment *n* = 1 RCTs without poor recruitment *n* = 5	1) Patient population and eligibility criteria: the RCT with poor recruitment focused on patients who did not fulfil the license criteria of rtPA (i.e., elderly patients > 80 years), whereas RCTs without poor recruitment did (focus on patients < 80 years)	The fact that the RCT with poor recruitment focused on elderly patients (> 80 years; i.e., narrow eligibility criteria, vulnerable patients) to be recruited in an acute care setting may have contributed to poor recruitment. The more complex subgroup hypotheses in the RCT with poor recruitment could have made the trial more difficult to explain to colleagues and patients. Finally, it is possible that the motivation of recruiters and patients was reduced, because evidence on the effectiveness from previous RCTs was already available (compromised equipoise)
	2) Target sample size: the number of patients planned to be enrolled was higher in the RCT with poor recruitment due to planned subgroup analyses (more complex design/hypotheses)	
	3) Publication chronology/available evidence: the RCT with poor recruitment (Sandercock et al. 2012) was the last RCT conducted for this research question to date	
#14: Transdermal nitroglycerin compared to placebo or usual care in laboring women (gestational age between 24 and 32 weeks) RCTs with poor recruitment *n* = 1 RCTs without poor recruitment *n* = 1	1) Patient burden during follow-up: longer follow-up with more complex assessments of the neonates in the RCT with poor recruitment	Laboring women in both RCTs had to be at high risk of preterm delivery but also not too advanced so that they could still be randomized; however, the RCT with poor recruitment imposed a higher burden on patients with longer follow-up and a more complex assessment of the neonates, probably reducing the motivation of patients and recruiters to participate; in contrast, the primary outcome (i.e., number of days from randomization to delivery) in the RCT without poor recruitment did not require any effort from patients. A few well-picked study centers may have been advantageous in this acute care setting. The motivation of recruiters and patients was probably further reduced in the trial with poor recruitment, when evidence on the effectiveness of the tested intervention (nitroglycerin) from other RCTs became available (compromised equipoise, Smith et al. 2007). Only 2 RCTs were considered in this group of RCTs, because the majority (*n* = 7) of RCTs included in the same systematic review did not report any sample size calculation/target sample size potentially hiding recruitment problems in these trials
	2) Primary outcome: the RCT with poor recruitment had a more complex primary outcome (neonatal morbidity associated with long-term morbidity and perinatal mortality vs. number of days from randomization to delivery)	
	3) Number of study centers: the RCT with poor recruitment had over 3 times more study centers than the RCT without poor recruitment	
	4) Publication chronology/available evidence: the RCT with poor recruitment was still ongoing when the RCT without poor recruitment was published	
#15: Vasopressin-containing regimen compared to epinephrine in cardiac arrest RCTs with poor recruitment *n* = 1 RCTs without poor recruitment *n* = 5	Number of study centers: the RCT with poor recruitment was a multicenter RCT whereas all but one RCT without poor recruitment (Gueugniaud et al. 2008) were single- center RCTs.	In this acute care setting with vulnerable patients a single center RCT may be advantageous due to higher motivation and better prepared/trained recruiters and trial team and closer monitoring of recruitment). The RCT with poor recruitment was conducted in 44 community-based emergency rooms. One other RCT without poor recruitment (Gueugniaud et al. 2008) had a similar number of study centers as the discontinued RCT, but authors reported that the network of centers was well stablished and experienced in running acute care RCTs.

*RCT* randomized controlled trial; All of the articles cited in Table 2 are referenced in Additional file 3

There was no consistent pattern as to whether an international or national multicenter setting or a single-center setting was advantageous for patient recruitment in RCTs. In research question #4, for instance, investigating antiarrhythmic drugs, the RCT with poor recruitment had three to four times fewer study centers than RCTs without poor recruitment; or in research area #2 (metastatic breast cancer therapy) the RCTs with poor recruitment were all restricted to a national setting, while the RCTs without poor recruitment were all done in large international collaborations. On the other hand, single-center RCTs or settings with only a few, carefully chosen study centers may have worked better in settings with particular logistical challenges (e.g., question #8 on primary angioplasty versus onsite thrombolysis and question #15 testing therapies for resuscitation) or the inclusion of particularly vulnerable patients (e.g., research questions #6 and #7 focusing on the recruitment of preterm neonates) in the absence of well-established and experienced trial networks.

We observed that investigator-sponsored RCTs are associated with a higher risk for poor recruitment than industry-sponsored RCTs; in research questions #1 and #12 all RCTs with poor recruitment were investigator-sponsored, while all RCTs without poor recruitment were industry-sponsored.

The chronology of RCTs, e.g., when a RCT is launched while other RCTs on the same research question have already been completed, appears to impact on recruitment. RCTs with poor recruitment were often initiated and published later, after other RCTs had already been successfully completed (research questions #2, #6, #10, #13, #14); i.e., evidence on the potential benefits and harms of a certain intervention was already available at some point during the conduct of such RCTs. This may have compromised the uncertainty about the tested treatments as an ethical precondition (equipoise), and the motivation of patients and recruiters for further randomization. In research questions #10 and #11 the control intervention(s) were already outdated, when the RCTs with poor recruitment were launched. In addition, in some RCTs with poor recruitment the burden for patients, such as numerous or invasive assessments during follow-up (research questions #1, #12, #14), or the burden for recruiters, such as the need to apply a complex scoring system in order to include patients (research question #9), was higher than in corresponding RCTs without poor recruitment. Furthermore, it happened that the tested interventions (already available drugs in RCTs with poor recruitment versus new drugs in RCTs without poor recruitment; research question #4) or the study design with side effects of the experimental drug being the primary outcome (research question #6) were less attractive in RCTs with poor recruitment than in corresponding RCTs without poor recruitment. If interventions to be compared in an RCT not only differed in the administered substance or drug but, in addition, in the route (e.g., intravenous or oral administration) or timing of application, then patients’ preferences could have compromised their willingness to be randomized (research question #11 in Table 2).

## Discussion

### Main findings

In this qualitative comparison between RCTs that did not achieve their originally planned sample size due to recruitment problems and RCTs that were completed as planned, we identified several reasons for poor recruitment. We found that RCTs with poor recruitment often had narrower eligibility criteria than RCTs without poor recruitment, were investigator-sponsored rather than industry-sponsored, and were less attractive for patients and recruiters due to higher burden, outdated control interventions, or already existing or accumulating evidence on benefits and harms of interventions from other RCTs. An existing network of study centers experienced in the conduct of RCTs is instrumental for successful recruitment, but whether one or a few study centers or an international multicenter design was more advantageous seemed to depend on the research context. With challenging settings such as acute care, vulnerable patients, or complex logistics, one or a few carefully chosen centers may be preferable due to closer monitoring of recruitment, potentially better prepared and motivated study staff, established procedures and, perhaps most important, efficient communication among the trial team. Prominent research areas in our sample were cancer research and research in acute care with, on average, more than one RCT with poor recruitment per research question suggesting that there are clusters of research areas typically prone to recruitment problems.

### Strengths and limitations

The strengths of our study include a new systematic approach to qualitatively compare RCTs discontinued or revised due to poor recruitment and RCTs completed as planned, that had previously been suggested [5] but, to our knowledge, never been used to date. We included 77 RCTs from a broad range of settings and research topics and analyzed them specifically in their context, thereby strengthening the applicability of our results.

Our study has several limitations. First, the present qualitative analysis was limited to the information provided in publications of RCTs and did not include information from other sources such as study protocols or interviews with trialists. This might have constituted a selection because the majority of RCTs discontinued for poor recruitment were not published in a peer-reviewed journal [1]. Second, we were not able to assess reasons for poor recruitment that were not described (e.g., lack of funding, a theme recurrently coming up in an interview study on the topic [4]). Third, we did not comprehensively search the literature for reports of RCTs discontinued or revised due to poor recruitment, but pragmatically started out with a sample of discontinued or revised RCTs identified in a previous study [1], and we used existing systematic and narrative reviews to find matching RCTs. In addition, we excluded 31 RCTs from our analysis because articles did not report a planned sample size, and therefore we were unable to judge whether the originally planned sample size was achieved or not. Fourth, the reporting of the patient recruitment process in included RCT publications provided little detail (irrespective of whether the RCT struggled with recruitment or not) compromising the effectiveness of our qualitative analysis. Particularly the fact that many articles did not report the number of patients screened for eligibility, the number not meeting eligibility criteria, and the number of patients declining to participate, often limited a better understanding of potential recruitment problems. Fifth, one researcher extracted all relevant information from included RCT publications and another checked this information rather than two researchers extracting relevant data independently and in duplicate. We chose this approach for feasibility reasons risking a higher rate of extraction errors. However, information directly relevant for our interpretation of reasons underlying recruitment problems were actually verified by two methodologically trained researchers. Sixth, although our study captured a broad range of clinical areas, 12 of the 15 research questions were related to drug therapy, leaving uncertainty whether our findings are equally applicable to other interventions such as surgery, behavior change, or complex interventions. Finally, although we found evidence for saturation in our qualitative analysis, the size of our study sample was mainly determined by practical issues of our approach.

### Comparison with other studies investigating poor recruitment in RCTs

Our results confirm several findings of previous studies using different methods. Investigator-sponsored (in the sense of investigator-initiated) RCTs, for instance, were found to be at higher risk for discontinuation due to poor recruitment than industry-sponsored RCTs by two studies using a quantitative approach with multivariable regression analysis [1, 2]. The common interpretation is that industry sponsorship is associated with sufficient funding and better planning and conduct, factors that facilitate successful patient recruitment. Moreover, the acute care setting (e.g., emergency rooms, intensive care units, care for preterm neonates) seems particularly prone to insufficient recruitment [10].

Similar to the present study, a systematic review of published reports of RCTs discontinued due to poor recruitment found that overly narrow eligibility criteria and prejudiced views of recruiters and patients on trial interventions were the most frequent reasons for poor recruitment [3]. Prejudiced views of patients and recruiters may come from different sources. Our study found that RCTs with poor recruitment were often launched relatively late in the sequence of RCTs on the same research question. As evidence accumulates over time, the uncertainty about the benefits and harms of a certain intervention or about the superiority of one intervention over another (equipoise) may become increasingly compromised. Some control interventions were even considered outdated right from the start of an RCT (research questions #10 and #11 in Table 2), which confirms the observation by Habre et al. [11]. In some instances, the route of application of an intervention or the more complex logistics associated with an intervention were less appealing to patients or recruiters. This is also consistent with the finding by Bernardez-Pereira et al. that single-arm clinical trials were less prone to discontinuation due to poor recruitment compared to multiple-arm trials. That is because in single-arm trials all patients receive the same intervention and, thus, are not confronted with the fact that they could be randomized to a different, maybe less preferred, treatment [2].

Another common finding in RCTs with poor recruitment was a high burden or inconvenience for patients or recruiters due to trial procedures (e.g., many follow-up visits, blood draws, lengthy questionnaires or case report forms). This was mentioned previously as a problem in several other qualitative studies [3, 12–14].

Our study highlights two aspects about recruitment challenges to RCTs that are, so far, not prominent in the published literature. First, investigators need to be aware of all other RCTs on a research question, their timing, and the accumulating evidence base, so that potentially compromising effects on the recruitment to their own trial can be minimized. Second, apart from the notion that well-established networks of collaborating study centers are an asset for the successful recruitment of patients to RCTs [4], it seems that a larger number of centers is not always advantageous. The performance of a study center typically depends on its commitment toward an RCT, the enthusiasm, training, and quality of communication of staff; therefore, challenging settings such as acute care, vulnerable patients, or complex logistics require careful selection and close monitoring of participating centers.

Finally, our study documents the urgent need for a more detailed reporting of participant recruitment in RCTs, which is in line with previous reports [3, 15, 16]. Furthermore, we did not find that poor reporting of the recruitment process was a particular issue of RCTs discontinued or revised for poor recruitment. Indicating that, if they do indeed get published, the reporting quality of the recruitment process is similarly poor as in RCTs that were completed as planned. The current Consolidated Standards of Reporting Trials (CONSORT) statement [17] explicitly recommends reporting the number of patients assessed for eligibility, number of eligible patients, and number of consenting patients. In the context of RCT discontinuation due to poor recruitment, however, investigators should additionally describe how they projected the number of eligible and consenting patients; whether a pilot study including informed consent was done; whether recruitment was closely monitored; which measures were undertaken to improve recruitment; and the specific root causes for recruitment failure in their case, so that future recruitment failures in that area of research can efficiently be prevented.

Given its magnitude and global presence of the problem, the evidence base on what actually works to improve recruitment in RCTs is still astonishingly thin [18, 19]. Based on the numerous analyses about the nature, extent, and causes for recruitment failure, it is time for international collaborative efforts to overcome the problem. Specifically, we need more randomized ‘studies within a trial’ (SWATs), i.e., promising interventions to improve recruitment need to be empirically evaluated within a host trial as propagated by the Trial Forge initiative, the Medical Research Council’s Network of Hubs for Trials Methodology Research in the UK, and the Health Research Board’s Trials Methodology Research Network in Ireland [20, 21].

## Conclusions

This qualitative comparison of RCTs discontinued or revised due to poor recruitment and RCTs completed as planned on the same research question complements previous efforts to identify risk factors for recruitment failure in RCTs, and to better understand the underlying mechanisms. Our study confirms previously identified causes such as narrow eligibility criteria, investigator-sponsorship, and a high burden of trial procedures for patients and recruiters, but also stresses the importance of considering the accumulating evidence and the timing of other RCTs on the same topic as well as carefully selecting and closely monitoring participating centers for RCTs in challenging settings. A more detailed reporting of patient recruitment in RCTs is urgently needed so that recruitment failure can provide lessons for other researchers in the future.

## Supplementary information

**Additional file 1.** Standards for Reporting Qualitative Research (SRQR)*

**Additional file 2.** Pre-specified checklist of items potentially associated with poor recruitment

**Additional file 3.** References of included randomized controlled trials (RCTs)

**Additional file 4.** Recruitment characteristics of included randomized controlled trials by research question

**Additional file 5.** Recruitment characteristics of included randomized controlled trials (RCTs)

**Additional file 6.** General characteristics of included randomized controlled trials (RCTs)

**Additional file 7.** Reporting quality of the recruitment process in included randomized controlled trials

## Data Availability

The data used for and/or analyzed during the current study is available in Additional files 1, 2, 3, 4, 5, 6, and 7.

## Abbreviations

CONSORT: Consolidated Standards of Reporting Trials
RCT: Randomized controlled trial
SRQR: Standards for Reporting Qualitative Research
SWAT: Study within a trial
