# Supplementary material for: Comparison of randomized controlled trials discontinued or revised for poor recruitment and completed trials with the same research question: a matched qualitative study
Source: Trials. 2019 Dec 30;20:800. doi: 10.1186/s13063-019-3957-4 (PMC6937940; doi:10.1186/s13063-019-3957-4)
Supplement: Supplementary file 2 — Additional file 2. Pre-specified checklist of items potentially associated with poor recruitment [file 13063_2019_3957_MOESM2_ESM.docx]

**APPENDIX B: Prespecified checklist of items potentially associated with poor recruitment**

| **Study ID** | **Who was the sponsor of the trial?** | **Was the trial design  unclear or complex?** | **Projection of recruitment based on a previous (complete or pilot) trial** | **Were recruitment support strategies considered?** | **Support from methods centre, clinical trial unit, contract research organization** | **Was the prevalence of the disease (prevalance of eligible patients at recruiting sites) included in sample size calculation?** | **Did the study test existing treatments vs new treatments?** | **Was the comparator an active intervention?** | **Were the target population vulnerable patients?** | **Were the target population uncommon patients?** | | **Was there any blinding of participants ?** | **Was there any blinding of care givers, including trialist ?** | | **Was there any blinding of outcome assessment ?** | **Allocation concealment** |
| --- | --- | --- | --- | --- | --- | --- | --- | --- | --- | --- | --- | --- | --- | --- | --- | --- |
| **1 Anvari2011**  **RCT with poor recruitment** | investigator | no, parallel design | No mention of a previous study used to plan recruitment | No recruitment support strategies reported | No logistical support of any third party reported | Not reported | Existing intervention | Active intervention | Not vulnerable | Common disease | | No | no | | no | non-blinded |
| 1 Galmiche2011  RCT without poor recruitment | industry | no, parallel design | No mention of a previous study used to plan recruitment | No recruitment support strategies reported | No logistical support of any third party reported | Not reported | Existing intervention | Active intervention | Not vulnerable | Common disease | | No | no | | no | unclear |
| **1 Grant2008**  **RCT with poor recruitment** | investigator | no, parallel design | No mention of a previous study used to plan recruitment | No recruitment support strategies reported | Logistical support of a third party was only involved in concealment of random allocation | Not reported | Existing intervention | Active intervention | Not vulnerable | Common disease | | No | No | | No | yes |
| 1 Mahon2004  RCT without poor recruitment | probably investigator | no, parallel design | No mention of a previous study used to plan recruitment | Contact by letter | No logistical support of any third party reported | Not reported | Existing intervention | Active intervention | Not vulnerable | Common disease | | no | unclear | | unclear | unclear |
| **2 Bonneterre2004**  **RCT with poor recruitment** | probably industry | no, parallel design | No mention of a previous study used to plan recruitment | No recruitment support strategies reported | No logistical support of any third party reported | Not reported | Existing intervention | Active intervention | Not vulnerable | Common disease | | no | high | | high | unclear |
| 2 Nabholtz2003  RCT without poor recruitment | industry | no, parallel design | No mention of a previous study used to plan recruitment | No recruitment support strategies reported | No logistical support of any third party reported | Not reported | Existing intervention | Active intervention | Not vulnerable | Common disease | | No | no | | no | unclear |
| **2 Bontenbal2005**  **RCT with poor recruitment** | industry | no, parallel design | No mention of a previous study used to plan recruitment | No recruitment support strategies reported | No logistical support of any third party reported | Not reported | Existing intervention | Active intervention | Not vulnerable | Common disease | | no | no | | no | yes |
| **2 Blohmer2010**  **RCT with poor recruitment** | industry | no, parallel design | No mention of a previous study used to plan recruitment | No recruitment support strategies reported | No logistical support of any third party reported | Not reported | Existing intervention | Active intervention | Not vulnerable | Common disease | | no | no | | no | yes |
| 2 Biganzoli2002  RCT without poor recruitment | industry | no, parallel design | No mention of a previous study used to plan recruitment | No recruitment support strategies reported | logistical support of a third party was only involved in concealment of random allocation | Not reported | Existing intervention | Active intervention | Not vulnerable | Common disease | | unclear | unclear | | unclear | yes |
| 2 Jassem2001  RCT without poor recruitment | industry | no, parallel design | No mention of a previous study used to plan recruitment | No recruitment support strategies reported | logistical support of a third party was only involved in concealment of random allocation | Not reported | Existing intervention | Active intervention | Not vulnerable | Common disease | | no | no | | no | yes |
| **3 Campos2009**  **RCT with poor recruitment** | industry |  | No mention of a previous study used to plan recruitment | No recruitment support strategies reported | No logistical support of any third party reported | Not reported | Existing intervention | Active intervention | Not vulnerable | Common disease | | no | no | | no | unclear |
| 3 Llombart-Cussac2012  RCT without poor recruitment | industry |  |  | No recruitment support strategies reported | No logistical support of any third party reported | Not reported | Existing intervention | Active intervention | Not vulnerable | Common disease | | no | no | | no | unclear |
| **4 Connolly2006**  **RCT with poor recruitment** | industry | no, parallel design | No mention of a previous study used to plan recruitment | No recruitment support strategies reported | No logistical support of any third party reported | Not reported | Existing intervention | Active intervention | Not vulnerable | Common disease | | no | no | | yes | yes |
| 4 Kowey2011  RCT without poor recruitment | industry | no, parallel design | No mention of a previous study used to plan recruitment | No recruitment support strategies reported | No logistical support of any third party reported | Not reported | New intervention | Active and Placebo | Not vulnerable | Common disease | | yes | yes | | unclear | yes |
| 4 Dorian2004  RCT without poor recruitment | industry | no, parallel design | No mention of a previous study used to plan recruitment | No recruitment support strategies reported | No logistical support of any third party reported | Not reported | New intervention | Active and Placebo | Not vulnerable | Common disease | | yes | yes | | yes | yes |
| **5 Dellinger2007**  **RCT with poor recruitment** | industry | no, parallel design | No mention of a previous study used to plan recruitment | No recruitment support strategies reported | ·       logistical support of a third party was only involved in concealment of random allocation | Not reported | Existing intervention | Placebo | alcohol and/or substance abuse | Common disease | | yes | yes | | yes | yes |
| **5 Rokke2007**  **RCT with poor recruitment** | probably investigator | no, parallel design | No mention of a previous study used to plan recruitment | No recruitment support strategies reported | No logistical support of any third party reported | Not reported | Existing intervention | No intervention in control arm, including usual care | alcohol and/or substance abuse | Common disease | | no | no | | no | unclear |
| **5 Garcia-Barrasa2009**  **RCT with poor recruitment** | investigator | no, parallel design | No mention of a previous study used to plan recruitment (a pilot study was planned within the same protocol for this trial) | No recruitment support strategies reported | No logistical support of any third party reported | Not reported | Existing intervention | Placebo | alcohol and/or substance abuse | Common disease | | yes | yes | | unclear | unclear |
| **6 Doyle2006**  **RCT with poor recruitment** | investigator | no, parallel design | No mention of a previous study used to plan recruitment | No recruitment support strategies reported | No logistical support of any third party reported | Not reported | Existing intervention | Placebo | Infants/ children | Common disease | no | | | yes | unclear | yes |
| 6 Brozanski 1995  RCT without poor recruitment | investigator | no, parallel design | No mention of a previous study used to plan recruitment | No recruitment support strategies reported | No logistical support of any third party reported | Not reported | Existing intervention | Placebo | Infants/ children | Common disease | yes | | | yes | yes | yes |
| 6 Durand 1995  RCT without poor recruitment | probably investigator | no, parallel design | No mention of a previous study used to plan recruitment | No recruitment support strategies reported | No logistical support of any third party reported | Not reported | Existing intervention | No intervention in control arm, including usual care | Infants/ children | Common disease | no | | | unclear | yes | unclear |
| 6 Cummings 1989  RCT without poor recruitment | probably investigator | no, parallel design | No mention of a previous study used to plan recruitment | No recruitment support strategies reported | No logistical support of any third party reported | The prevalence was estimated by an in house automated disease registry | Existing intervention | Placebo | Infants/ children | Common disease | yes | | | yes | yes | yes |
| **6 Kari 1993**  **RCT with poor recruitment** | investigator | no, parallel design | Recruitment was based on a national registry/national statistics | No recruitment support strategies reported | No logistical support of any third party reported | The prevalence was estimated by a national disease registry of the study country (the registry name was not reported) | Existing intervention | Placebo | Infants/ children | Common disease | unclear | | | yes | unclear | unclear |
| 6 Kovacs 1998  RCT without poor recruitment | probably investigator | no, parallel design | No mention of a previous study used to plan recruitment | No recruitment support strategies reported | No logistical support of any third party reported | Not reported | Existing intervention | Placebo | Infants/ children | Common disease | yes | | | yes | yes | unclear |
| 6 Ohlsson 1992  RCT without poor recruitment | investigator | no, parallel design | No mention of a previous study used to plan recruitment | No recruitment support strategies reported | No logistical support of any third party reported | Not reported | Existing intervention | sham | Infants/ children | Common disease | unclear | | | unclear | unclear | yes |
| 6 Walther 2003  RCT without poor recruitment | investigator | no, parallel design | No, but previous trials were used to assume an event rate | No recruitment support strategies reported | No logistical support of any third party reported | Not reported | Existing intervention | Placebo | Infants/ children | Common disease | unclear | | | yes | yes | yes |
| 6 Kazzi1990  RCT without poor recruitment | investigator | no, parallel design | No mention of a previous study used to plan recruitment | No recruitment support strategies reported | No logistical support of any third party reported | Not reported | Existing intervention | Placebo | Infants/ children | Common disease | no | | | yes | unclear | yes |
| **7 Field2005**  **RCT with poor recruitment** | probably investigator | no, parallel design | No mention of a previous study used to plan recruitment | No recruitment support strategies reported | No logistical support of any third party reported | Not reported | Existing intervention | Placebo | Infants/ children | Common disease | no | | | no | unclear | yes |
| 7 Kinsella 2006  RCT without poor recruitment | probably investigator | no, parallel design | No mention of a previous study used to plan recruitment | No recruitment support strategies reported | No logistical support of any third party reported | there was no description on how the prevalence of eligible patients was estimated | Existing intervention | Placebo | Infants/ children | Common disease | unclear | | | unclear | unclear | randomization numbers were linked to masked cylinders |
| 7 Schreiber2003  RCT without poor recruitment | investigator | yes, factorial design | No mention of a previous study used to plan recruitment | No recruitment support strategies reported | No logistical support of any third party reported | Not reported | Existing intervention | Placebo | Infants/ children | Common disease | unclear | | | unclear | yes | unclear |
| **7 Trial Group 1999***  **RCT with poor recruitment** | investigator | no, parallel design | No mention of a previous study used to plan recruitment | No recruitment support strategies reported | No logistical support of any third party reported | Not reported | Existing intervention | Placebo | Infants/ children | Common disease | probabaly not | | | no | no | yes |
| 7 Hascoet2005  RCT without poor recruitment | investigator | no, parallel design | No mention of a previous study used to plan recruitment | No recruitment support strategies reported | No logistical support of any third party reported | The prevalence was estimated based on a study done in a similar setting (prevalence of HRF) | Existing intervention | Placebo | Infants/ children | Common disease | unclear | | | unclear | unclear | yes |
| 7 Su and Chen2008  RCT without poor recruitment | investigator | no, parallel design | No mention of a previous study used to plan recruitment | No recruitment support strategies reported | No logistical support of any third party reported | Not reported | Existing intervention | Placebo | Infants/ children | Common disease | no | | | no | no | unclear |
| 7 Ballard2006  RCT without poor recruitment | investigator | no, parallel design | No mention of a previous study used to plan recruitment | No recruitment support strategies reported | No logistical support of any third party reported | Not reported | Existing intervention | Placebo | Infants/ children | Common disease | | yes | yes | | yes | yes |
| **8 Grines2002**  **RCT with poor recruitment** | probably investigator | no, parallel design | No mention of a previous study used to plan recruitment | No recruitment support strategies reported | No logistical support of any third party reported | Not reported | Existing intervention | No intervention in control arm, including usual care | Critically ill | Common disease | | no | no | | yes | yes |
| 8 Grines 1993  RCT without poor recruitment | probably investigator | no, parallel design | No mention of a previous study used to plan recruitment | No recruitment support strategies reported | No logistical support of any third party reported | Not reported | Existing intervention | No intervention in control arm, including usual care | Critically ill | Common disease | | unclear | unclear | | unclear | yes |
| 8 Le May2001  RCT without poor recruitment | probably investigator | no, parallel design | No mention of a previous study used to plan recruitment | No recruitment support strategies reported | No logistical support of any third party reported | Not reported | Existing intervention | No intervention in control arm, including usual care | Critically ill | Common disease | | unclear | unclear | | yes | yes |
| **8 Bonnefoy2002**  **RCT with poor recruitment** | probably investigator | no, parallel design | No mention of a previous study used to plan recruitment | No recruitment support strategies reported | No logistical support of any third party reported | Not reported | Existing intervention | No intervention in control arm, including usual care | Critically ill | Common disease | | unclear | unclear | | yes | yes |
| 8 Schömig2000  RCT without poor recruitment | probably investigator | no, parallel design | No mention of a previous study used to plan recruitment | No recruitment support strategies reported | No logistical support of any third party reported | Not reported | Existing intervention | No intervention in control arm, including usual care | Critically ill | Common disease | | unclear | unclear | | yes | unclear |
| **8 Aversano2002**  **RCT with poor recruitment** | probably investigator | no, parallel design | No mention of a previous study used to plan recruitment | No recruitment support strategies reported | logistical support of a third party involved throughout trial conduct | Not reported | Existing intervention | No intervention in control arm, including usual care | Critically ill | Common disease | | unclear | unclear | | yes | yes |
| **9 Höffken2007**  **RCT with poor recruitment** | industry | no, parallel design | No mention of a previous study used to plan recruitment | No recruitment support strategies reported | No logistical support of any third party reported | Not reported | Existing intervention | Active intervention | partly critically ill (10% mechanical ventilation) | Common disease | | no | no | | probably not | unclear |
| 9 Anzueto, 2006  RCT without poor recruitment | industry | no, parallel design | No mention of a previous study used to plan recruitment (a pilot study was planned within the same protocol for this trial) | No recruitment support strategies reported | logistical support of a third party involved in statistical analysis | Not reported | Existing intervention | Active intervention | Elderly | Common disease | | unclear | unclear | | unclear | unclear |
| **9 Ott, 2008**  **RCT with poor recruitment** | industry | no, parallel design | No mention of a previous study used to plan recruitment | No recruitment support strategies reported | logistical support of a third party partly involved in trial conduct | Not reported | Existing intervention | Active intervention | Critically ill | Common disease | | no | no | | unclear | yes |
| 9 Portier, 2005  RCT without poor recruitment | industry | no, parallel design | No mention of a previous study used to plan recruitment | No recruitment support strategies reported | No logistical support of any third party reported | Not reported | Existing intervention | Active intervention | Not vulnerable | Common disease | | no | no | | yes | yes |
| 9 Torres, 2008  RCT without poor recruitment | industry | no, parallel design | No mention of a previous study used to plan recruitment | No recruitment support strategies reported | ·       logistical support of a third party involved throughout trial conduct | Not reported | Existing intervention | Active intervention | Not vulnerable | Common disease | | yes | yes | | yes | unclear |
| 9 Welte, 2005  RCT without poor recruitment | industry | no, parallel design | No mention of a previous study used to plan recruitment | No recruitment support strategies reported | ·       logistical support of a third party partly involved in trial conduct (more than concealment of random allocation) | Not reported | Existing intervention | Active intervention | Not vulnerable | Common disease | | no | no | | unclear | yes |
| **10 Malmström2012**  **RCT with poor recruitment** | investigator | no, parallel design | No mention of a previous study used to plan recruitment | No recruitment support strategies reported | No logistical support of any third party reported | Not reported, only the prevelance of the primary outcome was estimated based on a previous study (pilot or published) | Existing intervention | Active intervention | Elderly | Common disease | | no | no | | unclear | yes |
| 10 Brada, 2010  RCT without poor recruitment | investigator | no, parallel design | No mention of a previous study used to plan recruitment | No recruitment support strategies reported | ·       logistical support of a third party was only involved in concealment of random allocation | Not reported | Existing intervention | Active intervention | Not vulnerable | Common disease | | no | no | | unclear; probably not | yes |
| 10 Stupp, 2005  RCT without poor recruitment | investigator | no, parallel design | No mention of a previous study used to plan recruitment | No recruitment support strategies reported | ·       logistical support of a third party partly involved in trial conduct (more than concealment of random allocation) | Not reported | Existing intervention | No intervention in control arm, including usual care | Not vulnerable | Common disease | | no | no | | unclear | yes |
| 10 Wick, 2012  RCT without poor recruitment | investigator | no, parallel design | No mention of a previous study used to plan recruitment |  | logistical support of a third party was only involved in concealment of random allocation | Not reported | Existing intervention | Active intervention | Elderly | Common disease | | no | no | | unclear; probably not | yes |
| **11 Pajk2008**  **RCT with poor recruitment** | industry | no, parallel design | No mention of a previous study used to plan recruitment | No recruitment support strategies reported | No logistical support of any third party reported | Not reported | Existing intervention | Active intervention | Not vulnerable | Common disease | | unclear | unclear | | unclear | unclear |
| **11 Bachelot, 2011**  **RCT with poor recruitment** | industry | no, parallel design | No mention of a previous study used to plan recruitment | No recruitment support strategies reported | logistical support of a third party partly involved in trial conduct | Not reported | Existing intervention | Active intervention | Not vulnerable | Common disease | | unclear | unclear | | unclear | unclear |
| 11 O`Shaughnessy JA, 2001  RCT without poor recruitment | industry | no, parallel design | No mention of a previous study used to plan recruitment | No recruitment support strategies reported | No logistical support of any third party reported | Not reported | New intervention | Active intervention | Not vulnerable | Common disease | | no | no | | yes | unclear |
| 11 O`Shaughnessy J, 2002  RCT without poor recruitment | industry | no, parallel design | No mention of a previous study used to plan recruitment | No recruitment support strategies reported | logistical support of a third party was only involved in concealment of random allocation | Not reported | New and existing | Active intervention | Not vulnerable | Common disease | | unclear | unclear | | yes | yes |
| 11 Mavroudis, 2010  RCT without poor recruitment | not reported | no, parallel design | No mention of a previous study used to plan recruitment | No recruitment support strategies reported | No logistical support of any third party reported | Not reported | Existing intervention | Active intervention | Not vulnerable | Common disease | | unclear | unclear | | unclear | yes |
| **11 Stockler, 2011**  **RCT with poor recruitment** | industry | no, parallel design | No mention of a previous study used to plan recruitment | No recruitment support strategies reported | No logistical support of any third party reported | Not reported | Existing intervention | Active intervention | Not vulnerable | Common disease | | unclear | unclear | | unclear | yes |
| 11 Stemmler, 2011  RCT without poor recruitment | industry | no, parallel design | No mention of a previous study used to plan recruitment | No recruitment support strategies reported | No logistical support of any third party reported | Not reported | Existing intervention | Active intervention | Not vulnerable | Common disease | | no | no | | no | unclear |
| **11 Talbot, 2002**  **RCT with poor recruitment** | industry | no, parallel design | No mention of a previous study used to plan recruitment | No recruitment support strategies reported | No logistical support of any third party reported | Not reported | ·       New_existing | Active intervention | Not vulnerable | Common disease | | no | no | | no | unclear |
| 11 Wardley, 2010  RCT without poor recruitment | industry | no, parallel design | No mention of a previous study used to plan recruitment | No recruitment support strategies reported | No logistical support of any third party reported | Not reported | Existing intervention | No intervention in control arm, including usual care | Not vulnerable | Common disease | | no | no | | no | unclear |
| **12 Perry2012**  **RCT with poor recruitment** | probably investigator | no, parallel design | No mention of a previous study used to plan recruitment | No recruitment support strategies reported | ·       logistical support of a third party was only involved in concealment of random allocation | Not reported | Existing intervention | Placebo | Not vulnerable | Common disease | | yes | yes | | yes | yes |
| **12 Sideras2006**  **RCT with poor recruitment** | investigator | no, parallel design | No mention of a previous study used to plan recruitment | No recruitment support strategies reported | logistical support of a third party was only involved in concealment of random allocation | Not reported | Existing intervention | No intervention in control arm, including usual care | Not vulnerable | Common disease | | yes at the beginning, no at the end of the study | yes at the beginning, no at the end of the study | | yes | yes |
| 12 Kakkar2004  RCT without poor recruitment | industry | no, parallel design | No mention of a previous study used to plan recruitment | No recruitment support strategies reported | No logistical support of any third party reported | Not reported, only the prevelance of the primary outcome was estimated based on a previous study (pilot or published) | Existing intervention | Placebo | Not vulnerable | Common disease | | unclear | unclear | | unclear | yes |
| 12 Agnelli2009  RCT without poor recruitment | industry | no, parallel design | No mention of a previous study used to plan recruitment | No recruitment support strategies reported | No logistical support of any third party reported | Not reported, only the prevelance of the primary outcome was estimated based on a previous study (pilot or published) | Existing intervention | Placebo | Not vulnerable | Common disease | | yes | yes | | yes | yes |
| **13 Sandercock2012**  **RCT with poor recruitment** | investigator | no, parallel design | No mention of a previous study used to plan recruitment | No recruitment support strategies reported | Third party support was mentioned at some place in the publication without any details | Not reported | Existing intervention | No intervention in control arm, including usual care | Critically ill | Common disease | | no | no | | yes | yes |
| 13 Hacke 1995  RCT without poor recruitment | industry | no, parallel design | No mention of a previous study used to plan recruitment | No recruitment support strategies reported | logistical support of a third party involved in data management and analysis | Not reported | Existing intervention | Placebo | Critically ill | Common disease | | unclear | unclear | | yes | yes |
| 13 Hacke 1998  RCT without poor recruitment | probably industry | no, parallel design | No mention of a previous study used to plan recruitment | No recruitment support strategies reported | logistical support of a third party involved throughout trial conduct | Not reported | Existing intervention | Placebo | Critically ill | Common disease | | unclear | unclear | | unclear | yes |
| 13 Hacke 2008  RCT without poor recruitment | industry | no, parallel design | No mention of a previous study used to plan recruitment | No recruitment support strategies reported | logistical support of a third party involved throughout trial conduct | Not reported | Existing intervention | Placebo | Critically ill | Common disease | | unclear | unclear | | yes | yes |
| 13 no authors listed ("…stroke study group"); NEJM 1995; part 2  RCT without poor recruitment | probably investigator | no, parallel design | No mention of a previous study used to plan recruitment | No recruitment support strategies reported | Third party support was mentioned at some place in the publication without any details ("Genentech…and monitored the sites") | Not reported | Existing intervention | Placebo | Critically ill | Common disease | | unclear | unclear | | yes | unclear |
| 13 Davis 2008  RCT without poor recruitment | investigator | no, parallel design | No mention of a previous study used to plan recruitment | No recruitment support strategies reported | No logistical support of any third party reported | Not reported | Existing intervention | Placebo | Critically ill | Common disease | | yes | yes | | yes | yes |
| **14 Smith2007**  **RCT with poor recruitment** | investigator | no, parallel design | No mention of a previous study used to plan recruitment | No recruitment support strategies reported | logistical support of a third party involved throughout trial conduct | Recruitment was based on a previous study in a similar setting; and the prevalance of the outcome of interest was based on a hospital database in the same setting | Existing intervention | Placebo | Pregnant women | Common disease | | yes | yes | | unclear | yes |
| 14 Bisits2004  RCT without poor recruitment | investigator | no, parallel design | No mention of a previous study used to plan recruitment | No recruitment support strategies reported | No logistical support of any third party reported | Not reported | New and existing | No intervention in control arm, including usual care | ·       Pregnant women | Common disease | | no | no | | unclear | yes |
| **15 Wenzel2004**  **RCT with poor recruitment** | investigator | no, parallel design | No mention of a previous study used to plan recruitment | No recruitment support strategies reported | No logistical support of any third party reported | mention of a previous study to assume effect | Existing intervention | Active intervention | Critically ill | Common disease | | unclear | unclear | | unclear | unclear |
| 15 Callaway 2006  RCT without poor recruitment | investigator | no, parallel design | no | ·       contact to community groups, public disclosure by newspaper, television, public forums, email site and call in telephone number | No logistical support of any third party reported | the prevalence of the primary outcome was estimated based on a previous study in the same setting | Existing intervention | Placebo | Critically ill | Common disease | | yes | yes | | yes | yes |
| 15 Mentzelopoulos 2009  RCT without poor recruitment | investigator | no, parallel design | no | No recruitment support strategies reported | No logistical support of any third party reported | Not reported | Existing intervention | Placebo | Critically ill | Common disease | | unclear | yes | | probably not | yes |
| 15 Lidner 1997  RCT without poor recruitment | investigator | no, parallel design | no | No recruitment support strategies reported | No logistical support of any third party reported | Not reported | Existing intervention | Active intervention | Critically ill | Common disease | | unclear | yes | | probably not | yes |
| 15 Gueugniaud 2008  RCT without poor recruitment | investigator | no, parallel design | no | No recruitment support strategies reported | No logistical support of any third party reported | Not reported | Existing intervention | Placebo | Critically ill | Common disease | | unclear | yes | | yes | yes |
| 15 Stiell 2001  RCT without poor recruitment | investigator | no, parallel design | no | No recruitment support strategies reported | No logistical support of any third party reported | the prevalence of the primary outcome was estimated based on a previous study in the same setting | Existing intervention | Existing intervention | Critically ill | Common disease | | unclear | yes | | unclear | yes |
