# Supplementary material for: Comparison of randomized controlled trials discontinued or revised for poor recruitment and completed trials with the same research question: a matched qualitative study
Source: Trials. 2019 Dec 30;20:800. doi: 10.1186/s13063-019-3957-4 (PMC6937940; doi:10.1186/s13063-019-3957-4)
Supplement: Supplementary file 3 — Additional file 3. References of included randomized controlled trials (RCTs) [file 13063_2019_3957_MOESM3_ESM.docx]

**APPENDIX C: References of included randomized controlled trials (RCTs)**

| **Included RCTs** | **Reference** |
| --- | --- |
| **1: Surgical treatment compared to proton-pump inhibitors in gastroesophageal reflux disease**  1 Anvari2011  RCT with poor recruitment  1 Galmiche2011  RCT without poor recruitment  1 Grant2008  RCT with poor recruitment  1 Mahon2004  RCT without poor recruitment | 1. Anvari M, Allen C, Marshall J, Armstrong D, Goeree R, Ungar W, et al. A randomized controlled trial of laparoscopic Nissen fundoplication versus proton pump inhibitors for the treatment of patients with chronic gastroesophageal reflux disease (GERD): 3-year outcomes. Surg Endosc. 2011;25(8):2547-54.  2. Galmiche JP, Hatlebakk J, Attwood S, Ell C, Fiocca R, Eklund S, et al. Laparoscopic antireflux surgery vs esomeprazole treatment for chronic GERD: the LOTUS randomized clinical trial. JAMA. 2011;305(19):1969-77.  3. Grant AM, Wileman SM, Ramsay CR, Mowat NA, Krukowski ZH, Heading RC, et al. Minimal access surgery compared with medical management for chronic gastro-oesophageal reflux disease: UK collaborative randomised trial. BMJ. 2008;337:a2664.  4. Mahon D, Rhodes M, Decadt B, Hindmarsh A, Lowndes R, Beckingham I, et al. Randomized clinical trial of laparoscopic Nissen fundoplication compared with proton-pump inhibitors for treatment of chronic gastro-oesophageal reflux. Br J Surg. 2005;92(6):695-9. |
| **2: Anthracyclines with or without any taxanes compared to anthracyclines plus any anti-cancer treatment or compared to single agent taxanes in metastatic breast cancer**  2 Bonneterre2004  RCT with poor recruitment  2 Nabholtz2003  RCT without poor recruitment  2 Bontenbal2005  RCT with poor recruitment  2 Blohmer2010  RCT with poor recruitment  2 Biganzoli2002  RCT without poor recruitment  2 Jassem2001  RCT without poor recruitment | 1. Biganzoli L, Cufer T, Bruning P, Coleman R, Duchateau L, Calvert AH, et al. Doxorubicin and paclitaxel versus doxorubicin and cyclophosphamide as first-line chemotherapy in metastatic breast cancer: The European Organization for Research and Treatment of Cancer 10961 Multicenter Phase III Trial. J Clin Oncol. 2002;20(14):3114-21.  2. Blohmer JU, Schmid P, Hilfrich J, Friese K, Kleine-Tebbe A, Koelbl H, et al. Epirubicin and cyclophosphamide versus epirubicin and docetaxel as first-line therapy for women with metastatic breast cancer: final results of a randomised phase III trial. Ann Oncol. 2010;21(7):1430-5.  3. Bonneterre J, Dieras V, Tubiana-Hulin M, Bougnoux P, Bonneterre ME, Delozier T, et al. Phase II multicentre randomised study of docetaxel plus epirubicin vs 5-fluorouracil plus epirubicin and cyclophosphamide in metastatic breast cancer. Br J Cancer. 2004;91(8):1466-71.  4. Bontenbal M, Creemers GJ, Braun HJ, de Boer AC, Janssen JT, Leys RB, et al. Phase II to III study comparing doxorubicin and docetaxel with fluorouracil, doxorubicin, and cyclophosphamide as first-line chemotherapy in patients with metastatic breast cancer: results of a Dutch Community Setting Trial for the Clinical Trial Group of the Comprehensive Cancer Centre. J Clin Oncol. 2005;23(28):7081-8.  5. Jassem J, Pienkowski T, Pluzanska A, Jelic S, Gorbunova V, Mrsic-Krmpotic Z, et al. Doxorubicin and paclitaxel versus fluorouracil, doxorubicin, and cyclophosphamide as first-line therapy for women with metastatic breast cancer: final results of a randomized phase III multicenter trial. J Clin Oncol. 2001;19(6):1707-15.  6. Nabholtz JM, Falkson C, Campos D, Szanto J, Martin M, Chan S, et al. Docetaxel and doxorubicin compared with doxorubicin and cyclophosphamide as first-line chemotherapy for metastatic breast cancer: results of a randomized, multicenter, phase III trial. J Clin Oncol. 2003;21(6):968-75. |
| **3: First line treatment with the aromatase inhibitor exemestane compared to anastrozole in postmenopausal women with advanced breast cancer**  3 Campos2009  RCT with poor recruitment  3 Llombart-Cussac2012  RCT without poor recruitment | 1. Campos SM, Guastalla JP, Subar M, Abreu P, Winer EP, Cameron DA. A comparative study of exemestane versus anastrozole in patients with postmenopausal breast cancer with visceral metastases. Clin Breast Cancer. 2009;9(1):39-44.  2. Llombart-Cussac A, Ruiz A, Anton A, Barnadas A, Antolin S, Ales-Martinez JE, et al. Exemestane versus anastrozole as front-line endocrine therapy in postmenopausal patients with hormone receptor-positive, advanced breast cancer Final Results from the Spanish Breast Cancer Group 2001-03 Phase 2 Randomized Trial. Cancer-Am Cancer Soc. 2012;118(1):241-7. |
| **4: Antiarrhythmic agents compared to placebo or background therapy with beta-blocker in patients with ventricular arrhythmia**  4 Connolly2006  RCT with poor recruitment  4 Kowey2011  RCT without poor recruitment  4 Dorian2004  RCT without poor recruitment | 1. Connolly SJ, Dorian P, Roberts RS, Gent M, Bailin S, Fain ES, et al. Comparison of beta-blockers, amiodarone plus beta-blockers, or sotalol for prevention of shocks from implantable cardioverter defibrillators: the OPTIC Study: a randomized trial. JAMA. 2006;295(2):165-71.  2. Dorian P, Borggrefe M, Al-Khalidi HR, Hohnloser SH, Brum JM, Tatla DS, et al. Placebo-controlled, randomized clinical trial of azimilide for prevention of ventricular tachyarrhythmias in patients with an implantable cardioverter defibrillator. Circulation. 2004;110(24):3646-54.  3. Kowey PR, Crijns HJGM, Aliot EM, Capucci A, Kulakowski P, Radzik D, et al. Efficacy and Safety of Celivarone, With Amiodarone as Calibrator, in Patients With an Implantable Cardioverter-Defibrillator for Prevention of Implantable Cardioverter-Defibrillator Interventions or Death The ALPHEE Study. Circulation. 2011;124(24):2649-U204. |
| **5: Prophylactic antibiotics compared to placebo or usual care in acute necrotising pancreatitis**  5 Dellinger2007  RCT with poor recruitment  5 Rokke2007  RCT with poor recruitment  5 Garcia-Barrasa2009  RCT with poor recruitment | 1. Dellinger EP, Tellado JM, Soto NE, Ashley SW, Barie PS, Dugernier T, et al. Early antibiotic treatment for severe acute necrotizing pancreatitis: a randomized, double-blind, placebo-controlled study. Ann Surg. 2007;245(5):674-83.  2. Garcia-Barrasa A, Borobia FG, Pallares R, Jorba R, Poves I, Busquets J, et al. A double-blind, placebo-controlled trial of ciprofloxacin prophylaxis in patients with acute necrotizing pancreatitis. J Gastrointest Surg. 2009;13(4):768-74.  3. Rokke O, Harbitz TB, Liljedal J, Pettersen T, Fetvedt T, Heen LO, et al. Early treatment of severe pancreatitis with imipenem: a prospective randomized clinical trial. Scand J Gastroenterol. 2007;42(6):771-6. |
| **6: Late postnatal (>7 days) corticosteroid treatment compared to placebo or usual care in preterm neonates**  6 Doyle2006  RCT with poor recruitment  6 Brozanski 1995  RCT without poor recruitment  6 Durand 1995  RCT without poor recruitment  6 Cummings 1989  RCT without poor recruitment  6 Kari 1993  RCT with poor recruitment  6 Kovacs 1998  RCT without poor recruitment  6 Ohlsson 1992  RCT without poor recruitment  6 Walther 2003  RCT without poor recruitment  6 Kazzi1990  RCT without poor recruitment | 1. Walther FJ, Findlay RD, Durand M. Adrenal suppression and extubation rate after moderately early low-dose dexamethasone therapy in very preterm infants. Early Hum Dev. 2003;74(1):37-45.  2. Ohlsson A, Calvert SA, Hosking M, Shennan AT. Randomized controlled trial of dexamethasone treatment in very-low-birth-weight infants with ventilator-dependent chronic lung disease. Acta Paediatr. 1992;81(10):751-6.  3. Kovacs L, Davis GM, Faucher D, Papageorgiou A. Efficacy of sequential early systemic and inhaled corticosteroid therapy in the prevention of chronic lung disease of prematurity. Acta Paediatr. 1998;87(7):792-8.  4. Kazzi NJ, Brans YW, Poland RL. Dexamethasone effects on the hospital course of infants with bronchopulmonary dysplasia who are dependent on artificial ventilation. Pediatrics. 1990;86(5):722-7.  5. Kari MA, Heinonen K, Ikonen RS, Koivisto M, Raivio KO. Dexamethasone treatment in preterm infants at risk for bronchopulmonary dysplasia. Arch Dis Child. 1993;68(5 Spec No):566-9.  6. Durand M, Sardesai S, McEvoy C. Effects of early dexamethasone therapy on pulmonary mechanics and chronic lung disease in very low birth weight infants: a randomized, controlled trial. Pediatrics. 1995;95(4):584-90.  7. Doyle LW, Davis PG, Morley CJ, McPhee A, Carlin JB, Investigators DS. Low-dose dexamethasone facilitates extubation among chronically ventilator-dependent infants: A multicenter, international, randomized, controlled trial. Pediatrics. 2006;117(1):75-83.  8. Cummings JJ, D'Eugenio DB, Gross SJ. A controlled trial of dexamethasone in preterm infants at high risk for bronchopulmonary dysplasia. N Engl J Med. 1989;320(23):1505-10.  9. Brozanski BS, Jones JG, Gilmour CH, Balsan MJ, Vazquez RL, Israel BA, et al. Effect of Pulse Dexamethasone Therapy on the Incidence and Severity of Chronic Lung-Disease in the Very-Low-Birth-Weight Infant. J Pediatr-Us. 1995;126(5):769-76. |
| **7: Ventilatory gas with nitric oxide compared to ventilatory gas without nitric oxide in preterm neonates**  7 Field2005  RCT with poor recruitment  7 Kinsella 2006  RCT without poor recruitment  7 Schreiber2003  RCT without poor recruitment  7 Trial Group 1999*  RCT with poor recruitment  7 Hascoet2005  RCT without poor recruitment  7 Su and Chen2008  RCT without poor recruitment  7 Ballard2006  RCT without poor recruitment | 1. Su PH, Chen JY. Inhaled nitric oxide in the management of preterm infants with severe respiratory failure. J Perinatol. 2008;28(2):112-6.  2. Schreiber MD, Gin-Mestan K, Marks JD, Huo DZ, Lee G, Srisuparp P. Inhaled nitric oxide in premature infants with the respiratory distress syndrome. New Engl J Med. 2003;349(22):2099-107.  3. Mercier JC, Thebaud B, Onody P, Storme L, van Overmeire B, Breart G, et al. Early compared with delayed inhaled nitric oxide in moderately hypoxaemic neonates with respiratory failure: a randomised controlled trial. Lancet. 1999;354(9184):1066-71.  4. Kinsella JP, Walsh WF, Bose CL, Gerstmann DR, Labella JJ, Sardesai S, et al. Inhaled nitric oxide in premature neonates with severe hypoxaemic respiratory failure: a randomised controlled trial. Lancet. 1999;354(9184):1061-5.  5. Hascoet JM, Fresson J, Claris O, Hamon I, Lombet J, Liska A, et al. The safety and efficacy of nitric oxide therapy in premature infants. J Pediatr-Us. 2005;146(3):318-23.  6. Field D, Elbourne D, Truesdale A, Grieve R, Hardy P, Fenton AC, et al. Neonatal ventilation with inhaled nitric oxide versus ventilatory support without inhaled nitric oxide for preterm infants with severe respiratory failure: The INNOVO multicentre randomised controlled trial (ISRCTN 17821339). Pediatrics. 2005;115(4):926-36.  7. Ballard RA, Truog WE, Cnaan A, Martin RJ, Ballard PL, Merrill JD, et al. Inhaled nitric oxide in preterm infants undergoing mechanical ventilation. New Engl J Med. 2006;355(4):343-53. |
| **8: Primary angioplasty compared to on site thrombolytic therapy in acute myocardial infarction (within 12 hours after onset)**  8 Grines2002  RCT with poor recruitment  8 Grines 1993  RCT without poor recruitment  8 Le May2001  RCT without poor recruitment  8 Bonnefoy2002  RCT with poor recruitment  8 Schömig2000  RCT without poor recruitment  8 Aversano2002  RCT with poor recruitment | 1. Schomig A, Kastrati A, Dirschinger J, Mehilli J, Schricke U, Pache J, et al. Coronary stenting plus platelet glycoprotein IIb/IIIa blockade compared with tissue plasminogen activator in acute myocardial infarction. Stent versus Thrombolysis for Occluded Coronary Arteries in Patients with Acute Myocardial Infarction Study Investigators. N Engl J Med. 2000;343(6):385-91.  2. Schomig A, Kastrati A, Dirschinger J, Mehilli J, Schricke U, Pache J, et al. Coronary stenting plus platelet glycoprotein IIb/IIIa blockade compared with tissue plasminogen activator in acute myocardial infarction. Stent versus Thrombolysis for Occluded Coronary Arteries in Patients with Acute Myocardial Infarction Study Investigators. N Engl J Med. 2000;343(6):385-91.  3. Le May MR, Labinaz M, Davies RF, Marquis JF, Laramee LA, O'Brien ER, et al. Stenting versus thrombolysis in acute myocardial infarction trial (STAT). J Am Coll Cardiol. 2001;37(4):985-91.  4. Grines CL, Westerhausen DR, Jr., Grines LL, Hanlon JT, Logemann TL, Niemela M, et al. A randomized trial of transfer for primary angioplasty versus on-site thrombolysis in patients with high-risk myocardial infarction: the Air Primary Angioplasty in Myocardial Infarction study. J Am Coll Cardiol. 2002;39(11):1713-9.  5. Grines CL, Browne KF, Marco J, Rothbaum D, Stone GW, O'Keefe J, et al. A comparison of immediate angioplasty with thrombolytic therapy for acute myocardial infarction. The Primary Angioplasty in Myocardial Infarction Study Group. N Engl J Med. 1993;328(10):673-9.  6. Bonnefoy E, Lapostolle F, Leizorovicz A, Steg G, McFadden EP, Dubien PY, et al. Primary angioplasty versus prehospital fibrinolysis in acute myocardial infarction: a randomised study. Lancet. 2002;360(9336):825-9. |
| **9: Moxifloxacin compared to other antibiotics in patients with pneumonia** 9 Höffken2007  RCT with poor recruitment  9 Anzueto, 2006  RCT without poor recruitment  9 Ott, 2008  RCT with poor recruitment  9 Portier, 2005  RCT without poor recruitment  9 Torres, 2008  RCT without poor recruitment  9 Welte, 2005  RCT without poor recruitment | 1. Anzueto A, Niederman MS, Pearle J, Restrepo MI, Heyder A, Choudhri SH, et al. Community-acquired pneumonia recovery in the elderly (CAPRIE): Efficacy and safety of moxifloxacin therapy versus that of levofloxacin therapy. Clin Infect Dis. 2006;42(1):73-81.  2. Hoffken G, Barth J, Rubinstein E, Beckmann H, group HAPs. A randomized study of sequential intravenous/oral moxifloxacin in comparison to sequential intravenous ceftriaxone/oral cefuroxime axetil in patients with hospital-acquired pneumonia. Infection. 2007;35(6):414-20.  3. Ott SR, Allewelt M, Lorenz J, Reimnitz P, Lode H, Grp GLAS. Moxifloxacin vs ampicillin/sulbactam in aspiration pneumonia and primary lung abscess. Infection. 2008;36(1):23-30.  4. Portier H, Brambilla C, Garre M, Paganin F, Poubeau P, Zuck P. Moxifloxacin monotherapy compared to amoxicillin-clavulanate plus roxithromycin for nonsevere community-acquired pneumonia in adults with risk factors. Eur J Clin Microbiol. 2005;24(6):367-76.  5. Torres A, Garau J, Arvis P, Carlet J, Choudhri S, Kureishi A, et al. Moxifloxacin monotherapy is effective in hospitalized patients with community-acquired pneumonia: The MOTIV study - A randomized clinical trial. Clin Infect Dis. 2008;46(10):1499-509.  6. Welte T, Petermann W, Schurmann D, Bauer TT, Reimnitz P, Grp MS. Treatment with sequential intravenous or oral moxifloxacin was associated with faster clinical improvement than was standard therapy for hospitalized patients with community-acquired pneumonia who received initial parenteral therapy. Clin Infect Dis. 2005;41(12):1697-705. |
| **10: Temozolomide (chemotherapeutic agent) alone or in combination with radiotherapy compared to no chemotherapy (e.g. radiotherapy), non-temozolomide based chemotherapy or temozolomide at different doses in glioma patients** 10 Malmström2012  RCT with poor recruitment  10 Brada, 2010  RCT without poor recruitment  10 Stupp, 2005  RCT without poor recruitment  10 Wick, 2012  RCT without poor recruitment | 1. Brada M, Stenning S, Gabe R, Thompson LC, Levy D, Rampling R, et al. Temozolomide Versus Procarbazine, Lomustine, and Vincristine in Recurrent High-Grade Glioma. Journal of Clinical Oncology. 2010;28(30):4601-8.  2. Malmstrom A, Gronberg BH, Marosi C, Stupp R, Frappaz D, Schultz H, et al. Temozolomide versus standard 6-week radiotherapy versus hypofractionated radiotherapy in patients older than 60 years with glioblastoma: the Nordic randomised, phase 3 trial. Lancet Oncol. 2012;13(9):916-26.  3. Stupp R, Mason WP, van den Bent MJ, Weller M, Fisher B, Taphoorn MJ, et al. Radiotherapy plus concomitant and adjuvant temozolomide for glioblastoma. N Engl J Med. 2005;352(10):987-96.  4. Wick W, Platten M, Meisner C, Felsberg J, Tabatabai G, Simon M, et al. Temozolomide chemotherapy alone versus radiotherapy alone for malignant astrocytoma in the elderly: the NOA-08 randomised, phase 3 trial. Lancet Oncol. 2012;13(7):707-15. |
| **11: Capecitabine-based chemotherapy compared to non-capecitabine chemotherapy in metastatic breast cancer** 11 Pajk2008  RCT with poor recruitment  11 Bachelot, 2011  RCT with poor recruitment  11 O`Shaughnessy, 2001  RCT without poor recruitment  11 O`Shaughnessy, 2002  RCT without poor recruitment  11 Mavroudis, 2010  RCT without poor recruitment  11 Stockler, 2011  RCT with poor recruitment  11 Stemmler, 2011  RCT without poor recruitment  11 Talbot, 2002  RCT with poor recruitment  11 Wardley, 2010  RCT without poor recruitment | 1. Bachelot T, Bajard A, Ray-Coquard I, Provencal J, Coeffic D, Agostini C, et al. Final Results of ERASME-4: A Randomized Trial of First-Line Docetaxel plus either Capecitabine or Epirubicin for Metastatic Breast Cancer. Oncology-Basel. 2011;80(3-4):262-8.  2. Mavroudis D, Papakotoulas P, Ardavanis A, Syrigos K, Kakolyris S, Ziras N, et al. Randomized phase III trial comparing docetaxel plus epirubicin versus docetaxel plus capecitabine as first-line treatment in women with advanced breast cancer. Ann Oncol. 2010;21(1):48-54.  3. O'Shaughnessy J, Miles D, Vukelja S, Moiseyenko V, Ayoub JP, Cervantes G, et al. Superior survival with capecitabine plus docetaxel combination therapy in anthracycline-pretreated patients with advanced breast cancer: phase III trial results. J Clin Oncol. 2002;20(12):2812-23.  4. Oshaughnessy JA, Blum J, Moiseyenko V, Jones SE, Miles D, Bell D, et al. Randomized, open-label, phase II trial of oral capecitabine (Xeloda) vs. a reference arm of intravenous CMF (cyclophosphamide, methotrexate and 5-fluorouracil) as first-line therapy for advanced/metastatic breast cancer. Ann Oncol. 2001;12(9):1247-54.  5. Pajk B, Cufer T, Canney P, Ellis P, Cameron D, Blot E, et al. Anti-tumor activity of capecitabine and vinorelbine in patients with anthracycline- and taxane-pretreated metastatic breast cancer: Findings from the EORTC 10001 randomized phase II trial. Breast. 2008;17(2):180-5.  6. Stemmler HJ, diGioia D, Freier W, Tessen HW, Gitsch G, Jonat W, et al. Randomised phase II trial of gemcitabine plus vinorelbine vs gemcitabine plus cisplatin vs gemcitabine plus capecitabine in patients with pretreated metastatic breast cancer. Brit J Cancer. 2011;104(7):1071-8.  7. Stockler MR, Harvey VJ, Francis PA, Byrne MJ, Ackland SP, Fitzharris B, et al. Capecitabine Versus Classical Cyclophosphamide, Methotrexate, and Fluorouracil As First-Line Chemotherapy for Advanced Breast Cancer. Journal of Clinical Oncology. 2011;29(34):4498-504.  8. Talbot DC, Moiseyenko V, Van Belle S, O'Reilly SM, Conejo EA, Ackland S, et al. Randomised, phase II trial comparing oral capecitabine (Xeloda (R)) with paclitaxel in patients with metastatic/advanced breast cancer pretreated with anthracyclines. Brit J Cancer. 2002;86(9):1367-72.  9. Wardley AM, Pivot X, Morales-Vasquez F, Zetina LM, Gaui MDD, Reyes DO, et al. Randomized Phase II Trial of First-Line Trastuzumab Plus Docetaxel and Capecitabine Compared With Trastuzumab Plus Docetaxel in HER2-Positive Metastatic Breast Cancer. Journal of Clinical Oncology. 2010;28(6):976-83. |
| **12: Primary thromboprophylaxis with heparin compared to placebo or usual care in ambulatory cancer patients receiving chemotherapy**  12 Perry2010  RCT with poor recruitment  12 Sideras2006  RCT with poor recruitment  12 Kakkar2004  RCT without poor recruitment  12 Agnelli2009  RCT without poor recruitment | 1. Agnelli G, Gussoni G, Bianchini C, Verso M, Mandala M, Cavanna L, et al. Nadroparin for the prevention of thromboembolic events in ambulatory patients with metastatic or locally advanced solid cancer receiving chemotherapy: a randomised, placebo-controlled, double-blind study. Lancet Oncol. 2009;10(10):943-9.  2. Kakkar AK, Levine MN, Kadziola Z, Lemoine NR, Low V, Patel HK, et al. Low molecular weight heparin, therapy with dalteparin, and survival in advanced cancer: The fragmin advanced malignancy outcome study (FAMOUS). Journal of Clinical Oncology. 2004;22(10):1944-8.  3. Perry JR, Julian JA, Laperriere NJ, Geerts W, Agnelli G, Rogers LR, et al. PRODIGE: a randomized placebo-controlled trial of dalteparin low-molecular-weight heparin thromboprophylaxis in patients with newly diagnosed malignant glioma. J Thromb Haemost. 2010;8(9):1959-65.  4. Sideras K, Schaefer PL, Okuno SH, Sloan JA, Kutteh L, Fitch TR, et al. Low-molecular-weight heparin in patients with advanced cancer: A phase 3 clinical trial. Mayo Clin Proc. 2006;81(6):758-67. |
| **13: Recombinant tissue plasminogen activator compared to placebo in acute ischemic stroke** 13 Sandercock2012  RCT with poor recruitment  13 Hacke 1995  RCT without poor recruitment  13 Hacke 1998  RCT without poor recruitment  13 Hacke 2008  RCT without poor recruitment  13 Stroke study group  RCT without poor recruitment  13 Davis 2008  RCT without poor recruitment | 1. Davis SM, Donnan GA, Parsons MW, Levi C, Butcher KS, Peeters A, et al. Effects of alteplase beyond 3 h after stroke in the Echoplanar Imaging Thrombolytic Evaluation Trial (EPITHET): a placebo-controlled randomised trial. Lancet Neurol. 2008;7(4):299-309.  2. Hacke W, Kaste M, Bluhmki E, Brozman M, Davalos A, Guidetti D, et al. Thrombolysis with alteplase 3 to 4.5 hours after acute ischemic stroke. New Engl J Med. 2008;359(13):1317-29.  3. Hacke W, Kaste M, Fieschi C, Toni D, Lesaffre E, Vonkummer R, et al. Intravenous Thrombolysis with Recombinant Tissue-Plasminogen Activator for Acute Hemispheric Stroke - the European Cooperative Acute Stroke Study (Ecass). Jama-J Am Med Assoc. 1995;274(13):1017-25.  4. Hacke W, Kaste M, Fieschi C, von Kummer R, Davalos A, Meier D, et al. Randomised double-blind placebo-controlled trial of thrombolytic therapy with intravenous alteplase in acute ischaemic stroke (ECASS II). Lancet. 1998;352(9136):1245-51.  5. Marler JR, Brott T, Broderick J, Kothari R, Odonoghue M, Barsan W, et al. Tissue-Plasminogen Activator for Acute Ischemic Stroke. New Engl J Med. 1995;333(24):1581-7.  6. Sandercock P, Wardlaw JM, Lindley RI, Dennis M, Cohen G, Murray G, et al. The benefits and harms of intravenous thrombolysis with recombinant tissue plasminogen activator within 6 h of acute ischaemic stroke (the third international stroke trial [IST-3]): a randomised controlled trial. Lancet. 2012;379(9834):2352-63. |
| **14: Transdermal nitroglycerin compared to placebo or usual care in laboring women (gestational age between 24 and 32 weeks)**  14 Smith2007  RCT with poor recruitment  14 Bisits2004  RCT without poor recruitment | 1. Bisits A, Madsen G, Knox M, Gill A, Smith R, Yeo G, et al. The Randomized Nitric Oxide Tocolysis Trial (RNOTT) for the treatment of preterm labor. Am J Obstet Gynecol. 2004;191(3):683-90.  2. Smith GN, Walker MC, Ohlsson A, O'Brien K, Windrim R, Canadian Preterm Labour Nitroglycerin Trial G. Randomized double-blind placebo-controlled trial of transdermal nitroglycerin for preterm labor. Am J Obstet Gynecol. 2007;196(1):37 e1-8. |
| **15: Vasopressin containing regimen compared to epinephrine in cardiac arrest**  15 Wenzel2004  RCT with poor recruitment  15 Callaway 2006  RCT without poor recruitment  15 Mentzelopoulos 2009  RCT without poor recruitment  15 Lidner 1997  RCT without poor recruitment  15 Gueugniaud 2008  RCT without poor recruitment  15 Stiell 2001  RCT without poor recruitment | 1. Callaway CW, Hostler D, Doshi AA, Pinchalk M, Roth RN, Lubin J, et al. Usefulness of vasopressin administered with epinephrine during out-of-hospital cardiac arrest. Am J Cardiol. 2006;98(10):1316-21.  2. Gueugniaud PY, David JS, Chanzy E, Hubert H, Dubien PY, Mauriaucourt P, et al. Vasopressin and epinephrine vs. epinephrine alone in cardiopulmonary resuscitation. N Engl J Med. 2008;359(1):21-30.  3. Lindner KH, Dirks B, Strohmenger HU, Prengel AW, Lindner IM, Lurie KG. Randomised comparison of epinephrine and vasopressin in patients with out-of-hospital ventricular fibrillation. Lancet. 1997;349(9051):535-7.  4. Mentzelopoulos SD, Zakynthinos SG, Tzoufi M, Katsios N, Papastylianou A, Gkisioti S, et al. Vasopressin, epinephrine, and corticosteroids for in-hospital cardiac arrest. Arch Intern Med. 2009;169(1):15-24.  5. Stiell IG, Hebert PC, Wells GA, Vandemheen KL, Tang AS, Higginson LA, et al. Vasopressin versus epinephrine for inhospital cardiac arrest: a randomised controlled trial. Lancet. 2001;358(9276):105-9.  6. Wenzel V, Krismer AC, Arntz HR, Sitter H, Stadlbauer KH, Lindner KH, et al. A comparison of vasopressin and epinephrine for out-of-hospital cardiopulmonary resuscitation. N Engl J Med. 2004;350(2):105-13. |
