# Supplementary material for: Comparison of randomized controlled trials discontinued or revised for poor recruitment and completed trials with the same research question: a matched qualitative study
Source: Trials. 2019 Dec 30;20:800. doi: 10.1186/s13063-019-3957-4 (PMC6937940; doi:10.1186/s13063-019-3957-4)
Supplement: Supplementary file 4 — Additional file 4. Recruitment characteristics of included randomized controlled trials by research question [file 13063_2019_3957_MOESM4_ESM.docx]

**APPENDIX D: Recruitment characteristics of included randomized controlled trials by research question**

| **Matched studies with the same research question** | **Planned target number of patients (range)** | **Number of patients assessed for eligibility (range)** | **Number of patients randomized (range)** | **Start of patient recruitment (range)** | **Duration of recruitment period (range)** | **Network of recruiting centers (number of study centers, range)** | **Number of patients recruited per year and center* (range)** | **Trial initiator (funding source)** |  |
| --- | --- | --- | --- | --- | --- | --- | --- | --- | --- |
| **1: Surgical treatment compared to proton-pump inhibitors in gastroesophageal reflux disease** | | | | | | | | | |
| RCT with poor recruitment n=2 | 216-600 | 1078-1666 | 104-357 | Oct 00 – Mar 01 | 3y 3m – 3y 11m | National (1-21) | 5.2-26.6 | Investigator (public) |  |
| RCT without poor recruitment n=2 | 215-550 | 626 (NR, n=1) | 217-554 | Jul 97 – Oct 01 | 4y – 4y 1m | National (2)/ international (NR) | 26.6 (Not estimable, n=1) | Industry (industry) |  |
| **2: Anthracyclines with or without any taxanes compared to anthracyclines plus any anti-cancer treatment or compared to single agent taxanes in metastatic breast cancer** | | | | | | | | | |
| RCT with poor recruitment n=3 | 200-346 | NR | 142-240 | Mar 97 Feb 00 | 2y 2m – 5y 1m | National (12-49) | 1.3-5.5 | Industry (industry) |  |
| RCT without poor recruitment n=3 | 260-428 | NR | 267-429 | Nov 96 – Nov 96 (NR, n=1) | 2y 5m – 3y 3m (NR, n=1) | International (29-58) | 6.5 (Not estimable, n=2) | Industry (industry, NR, n=1) |  |
| **3: First line treatment with the aromatase inhibitor exemestane compared to anastrozole in postmenopausal women with advanced breast cancer** | | | | | | | | | |
| RCT with poor recruitment n=1 | 216 | NR | 130 | Nov 00 | 4y 1m | International (39) | 0.8 | Industry (industry) |  |
| RCT without poor recruitment n=1 | 100 | NR | 103 | Sep 01 | 1y 6m | National (13) | 5.3 | Industry (industry) |  |
| **4: Antiarrhythmic agents compared to placebo or background therapy with beta-blocker in patients with ventricular arrhythmia** | | | | | | | | | |
| RCT with poor recruitment n=1 | 700 | NR | 412 | Jan 01 | 3y 8m | International (39) | 2.9 | Industry (industry) |  |
| RCT without poor recruitment n=2 | 486-624 | NR | 486-633 | Sep 01 – Sep 09 | 1y 6m – 1y 8m | International (129-151) | 1.9-3.3 | Industry (industry) |  |
| **5: Prophylactic antibiotics compared to placebo or usual care in acute necrotising pancreatitis** | | | | | | | | | |
| RCT with poor recruitment n=3 | 134-240 | 798-807 (NR, n=1) | 46 - 100 | 97 (month not reported) – Feb 03 | 1y 10m – 5y | National (1-7)/ international (32) | 1.7-12.8 | Industry/ investigator (industry/internalfunding source) |  |
| RCT without poor recruitment n=0 | - | - | - | - | - | - | - | - |  |
| **6: Late postnatal (>7 days) corticosteroid treatment compared to placebo or usual care in preterm neonates** | | | | | | | | | |
| RCT with poor recruitment n=2 | 79 - 814 | NR | 41-70 | Jan 89 – Mar 00 | 2y 1m – 2y 7m | International/ national (1-11) | 2.5-4.9 | Investigator (public) |  |
| RCT without poor recruitment n=7 | 22 - 76 | 173 - 356 (Not reported, n=4) | 23 - 88 | Jan 86 – Mar 93 (NR, n=2) | 1y 6m – 3y | National (1) | 9.2-44.0 | Investigator (public, NR, n=3) |  |
| **7: Ventilatory gas with nitric oxide compared to ventilatory gas without nitric oxide in preterm neonates** | | | | | | | | | |
| RCT with poor recruitment n=2 | 200 - 360 | NR | 108-204 | Apr 95 – Feb 97 | 2y 2m – 4y 10m | International (26-34) | 0.7-3.6 | Investigator (public funding, not reported n=1) |  |
| RCT without poor recruitment n=5 | 64 - 814 | 936-5129 (NR, n=2) | 65-860 | Oct 98 – March 01 | 1y 7m – 6 y | International (10)/ national (1-21) | 5.7-69.0 | Investigator (industry/public/mix) |  |
| **8: Primary angioplasty compared to on site thrombolytic therapy in acute myocardial infarction (within 12 hours after onset)** | | | | | | | | | |
| RCT with poor recruitment n=3 | 430 - 2550 | 548 (NR, n=2) | 138-840 | Jul 96 – Jun 97 (NR, n=2) | 2y 11m – 3y 3m | International (12)/ national (11-27) | 3.5-14.1 | Investigator (public/industry) |  |
| RCT without poor recruitment n=3 | 124 - 370 | NR | 123-395 | Jun 90 – Dec 97 | 1y 2m – 1y 10m | International (NR)/ national (1, NR, n=1) | 67.1 (Not estimable, n=2) | Investigator (public/industry) |  |
| **9: Moxifloxacin compared to other antibiotics in patients with pneumonia** | | | | | | | | | |
| RCT with poor recruitment n=2 | 154 - 566 | NR | 139-161 | May 00 – Feb 01 | 1y 9m – 3y 11m | International (39)/ national (15) | 2.4-2.4 | Industry (public/industry) |  |
| RCT without poor recruitment n=4 | 278 - 608 | 432 (NR, n=3) | 349-733 | Feb 01 – Jan 04 | 1y 2m – 1y 10m | International (54-69)/ national (47-80) | 2.4-7.5 (Not estimable, n=1) | Industry (industry) |  |
| **10: Temozolomide (chemotherapeutic agent) alone or in combination with radiotherapy compared to no chemotherapy (e.g. radiotherapy), non-temozolomide based chemotherapy or temozolomide at different doses in glioma patients** | | | | | | | | | |
| RCT with poor recruitment n=1 | 480 | NR | 342 | Feb 00 | 9y 4m | International (28) | 1.3 | Investigator (public) |  |
| RCT without poor recruitment n=3 | 382 - 500 | 584 (NR, n=2) | 412-573 | Aug 00 – May 05 | 1y 6m – 4y 7m | International (24-85)/national (36) | 2.7-4.5 | Investigator (public/industry) |  |
| **11: Capecitabine-based chemotherapy compared to non-capecitabine chemotherapy in metastatic breast cancer** | | | | | | | | | |
| RCT with poor recruitment n=4 | 72 - 465 | NR | 42-325 | May 96 – Apr 04 | 10m – 3y 11m | International (8-18)/ national (15, NR, n=1 (34) | 1.6-2.8 | Industry (public/industry) |  |
| RCT without poor recruitment n=5 | 85 - 500 | 307 (NR, n=4) | 95-511 | May 96 – 2003 (month not reported) (NR, n=1) | 1y 0m – 5y 6m | International (23-75)/ national (24; NR, n=1), NR, n=1 | 1.5-4.1 (Not estimable, n=2) | Industry (industry), NR, n=1 |  |
| **12: Primary thromboprophylaxis with heparin compared to placebo or usual care in ambulatory cancer patients receiving chemotherapy** | | | | | | | | | |
| RCT with poor recruitment n=2 | 512 - 530 | 563 (NR, n=1) | 141-186 | Dec 98 – Oct 02 | 2y 6m – 3y 7m n=1) | International (15)/ national (15, NR, n=1) | 3.5 (Not estimable, n=1) | Investigator (public/industry) |  |
| RCT without poor recruitment n=2 | 366 - 1080 | NR | 385-1166 | May 95 – Oct 03 | 3y 7m – 5y 11m | International (10)/ national (62) | 5.2-6.5 | Industry (industry) |  |
| **13: Recombinant tissue plasminogen activator compared to placebo in acute ischemic stroke** | | | | | | | | | |
| RCT with poor recruitment n=1 | 6000 | NR | 3035 | May 00 | 11y 2m | International (156) | 1.7 | Investigator (public) |  |
| RCT without poor recruitment n=5 | 100 - 800 | 814 - 3908 (NR, n=3) | 101-821 | 1992 (month not reported) – Jul 03 | 1y 3m – 5y 9m | International 15-130)/ national (NR) | 1.2 – 7.1 (Not estimable, n=1) | Industry/ investigator (public/industry, not reported n =1)) |  |
| **14: Transdermal nitroglycerin compared to placebo or usual care in laboring women (gestational age between 24 and 32 weeks)** | | | | | | | | | |
| RCT with poor recruitment n=1 | 600 | NR | 158 | May 01 | 3y 1m | National (14) | 3.7 | Investigator (public) |  |
| RCT without poor recruitment n=1 | 240 | 1174 | 238 | Apr 97 | 3y 1m | International (4) | 19.3 | Investigator (public) |  |
| **15: Vasopressin containing regimen compared to epinephrine in cardiac arrest** | | | | | | | | | |
| RCT with poor recruitment n=1 | 1500 | 5967 | 1219 | Jun 99 | 2y 9m | International (44) | 10.1 | Investigator (public/industry) |  |
| RCT without poor recruitment n=5 | 38 - 2416 | 139-701 (NR, n=2) | 40-2956 | Jul 94 – Jun 06 | 9m – 1y11m | International (3)/ national (1-31) | 49.8-300 | Investigator (public) |  |

Abbreviations: RCT, randomized clinical trial; NR, Not reported; y, year; m, month; *rough estimate for recruitment speed based on own calculations (number of patients recruited devided by recruitment duration in years, devided by number of study centers); not adjusted to the time a site was actually open for recruitment, because this was not reported in the publications of the included RCTs.
