# Supplementary material for: Comparison of randomized controlled trials discontinued or revised for poor recruitment and completed trials with the same research question: a matched qualitative study
Source: Trials. 2019 Dec 30;20:800. doi: 10.1186/s13063-019-3957-4 (PMC6937940; doi:10.1186/s13063-019-3957-4)
Supplement: Supplementary file 5 — Additional file 5. Recruitment characteristics of included randomized controlled trials (RCTs) [file 13063_2019_3957_MOESM5_ESM.docx]

**APPENDIX E: Recruitment characteristics of included randomized controlled trials (RCTs)**

| **Study ID** | **n patients planned to enroll** | **n patients assessed for eligibility** | | **n patients randomized** | | **Start of recruitment period** | | **Duration of recruitment period** | | **Network of recruiting centers** | | **Number of study centers** | **n patients recruited per year and center*** | **Who conducted the recruitment?** | | **Were recruitment support strategies considered?** | **funding source** | **sponsor** | |
| --- | --- | --- | --- | --- | --- | --- | --- | --- | --- | --- | --- | --- | --- | --- | --- | --- | --- | --- | --- |
| **1 Anvari2011**  **RCT with poor recruitment** | **216** | **1666** | | **104** | | **Oct 2000** | | **3y 11 m** | | **national** | | **1** | **26.6** | **not reported** | | **not reported** | **public** | **investigator** | |
| 1 Galmiche2011  RCT without poor recruitment | 550 | 626 | | 554 | | Oct 2001 | | 4 y | | international | | not reported | not estimable | not reported | | not reported | industry | industry | |
| **1 Grant2008**  **RCT with poor recruitment** | **600** | **1078** | | **357** | | **Mar 01** | | **3 y 3 m** | | **national** | | **21** | **5.2** | **study nurses** | | **not reported** | **public** | **investigator** | |
| 1 Mahon2004  RCT without poor recruitment | 215 | not reported | | 217 | | Jul 97 | | 4 y 1 m | | national | | 2 | 26.6 | primary care physician | | Contact by letter | partly industry | probably investigator | |
| **2 Bonneterre2004**  **RCT with poor recruitment** | **200** | **not reported** | | **142** | | **Sep 98** | | **2 y 2 m** | | **national** | | **12** | **5.5** | **not reported** | | **not reported** | **not reported** | **industry** | |
| 2 Nabholtz2003  RCT without poor recruitment | 428 | not reported | | 429 | | not reported | | not reported | | international | | 58 | not estimable | Not reported | | not reported | industry | industry | |
| **2 Bontenbal2005**  **RCT with poor recruitment** | **260** | **not reported** | | **216** | | **Mar 97** | | **5 y 1 m** | | **national** | | **19** | **2.2** | **not reported** | | **not reported** | **industry** | **industry** | |
| **2 Blohmer2010**  **RCT with poor recruitment** | **346** | **not reported** | | **240** | | **Feb 00** | | **3 y 9 m** | | **national** | | **49** | **1.3** | **not reported** | | **not reported** | **industry** | **industry** | |
| 2 Biganzoli2002  RCT without poor recruitment | 260 | Not reported | | 275 | | Nov 96 | | 3 y 3 m | | not reported | | not reported | not estimable | not reported | | not reported | partly industry | industry | |
| 2 Jassem2001  RCT without poor recruitment | 260 | not reported | | 267 | | Nov 96 | | 2 y 5 m | | international | | 29 | 6.5 | not reported | | not reported | industry | industry | |
| **3 Campos2009**  **RCT with poor recruitment** | **216** | **not reported** | | **130** | | **Nov 00** | | **4 y 1 m** | | **international** | | **39** | **0.8** | **not reported** | | **not reported** | **industry** | **industry** | |
| 3 Llombart-Cussac2012  RCT without poor recruitment | 100) | not reported | | 103 | | Sep 01 | | 1 y 6 m | | national | | 13 | 5.3 | not reported | | not reported | industry | industry | |
| **4 Connolly2006**  **RCT with poor recruitment** | **700** | **NR** | | **412** | | **Jan 01** | | **3 y 8 m** | | **international** | | **39** | **2.9** | **NR** | | **not reported** | **industry** | **industry** | |
| 4 Kowey2011  RCT without poor recruitment | 486 | NR | | 486 | | Sep 09 | | 1,5 | | international | | 151 | 1.9 | NR | | not reported | industry | industry | |
| 4 Dorian2004  RCT without poor recruitment | 624 | NR | | 633 | | Sep 01 | | 1,5 | | international | | 129 | 3.3 | NR | | not reported | industry | industry | |
| **5 Dellinger2007**  **RCT with poor recruitment** | **240** | **807** | | **100** | | **Feb 03** | | **1 y 10 m** | | **international** | | **32** | **1.7** | **not reported** | | **not reported** | **industry** | **industry** | |
| **5 Rokke2007**  **RCT with poor recruitment** | **160** | **not reported** | | **73** | | **1997 (month not reported)** | | **5 y** | | **national** | | **7** | **2.1** | **not reported** | | **not reported** | **partly industry** | **probably investigator** | |
| **5 Garcia-Barrasa2009**  **RCT with poor recruitment** | **134** | **798** | | **46** | | **May 99** | | **national** | | **1** | | **12.8** | **not reported** | **not reported** | | **not reported** | **internal (in house) funding** | **investigator** | |
| **6 Doyle2006**  **RCT with poor recruitment** | **814** | **not reported** | | **70** | | **Mar 00** | | **2 y 7 m** | | **international** | | **11** | **2.5** | | **not reported** | **not reported** | **public** | **investigator** |  |
| 6 Brozanski 1995  RCT without poor recruitment | 76 | 356 | | 88 | | Mar 91 | | 2 y | | national | | 1 | 44.0 | | not reported | not reported | public | investigator |  |
| 6 Durand 1995  RCT without poor recruitment | 40 | not reported | | 44 | | not reported | | ca. 2 y** | | national | | 1 | 22.0 | | not reported | not reported | not reported | probably investigator |  |
| 6 Cummings 1989  RCT without poor recruitment | 30 | not reported | | 36 | | Jan 86 | | 1 y 6 m | | national | | 1 | 24.0 | | not reported | not reported | not reported | probably investigator |  |
| **6 Kari 1993**  **RCT with poor recruitment** | **79** | **not reported** | | **41** | | **Jan 89** | | **2 y 1 m** | | **national** | | **4** | **4.9** | | **not reported** | **not reported** | **public** | **investigator** |  |
| 6 Kovacs 1998  RCT without poor recruitment | 60 | 173 | | 60 | | Mar 93 | | 2 y 7 m | | national | | 1 | 23.2 | | not reported | not reported | not reported | probably investigator |  |
| 6 Ohlsson 1992  RCT without poor recruitment | 22 | 309 | | 25 | | Apr 86 | | 2 y 2m | | national | | 1 | 11.5 | | not reported | not reported | public | investigator |  |
| 6 Walther 2003  RCT without poor recruitment | 28 | not reported | | 36 | | not reported | | 3 y | | national | | 1 | 12.0 | | not reported | not reported | public | investigator |  |
| 6 Kazzi1990  RCT without poor recruitment | 22 | not reported | | 23 | | Aug 86 | | 2 y 6 m | | national | | 1 | 9.2 | | not reported | not reported | not reported | investigator |  |
| **7 Field2005**  **RCT with poor recruitment** | **200** | | **not reported** | | **108** | | **Feb 97** | | **international** | | **34** | **0.7** | **not reported** | | **not reported** | **not reporrted** | **not reported** | **probably investigator** |  |
| 7 Kinsella 2006  RCT without poor recruitment | 792 | 4562 | | 793 | | March 01 | | 4 y 3 m | | national | | 17 | 11.0 | not reported | | not reported | public | probably investigator | |
| 7 Schreiber2003  RCT without poor recruitment | 200 | not reported | | 207 | | Oct 98 | | 3 y | | national | | 1 | 69.0 | not reported | | not reported | industry | investigator | |
| **7 Trial Group 1999**  **RCT with poor recruitment** | **360** | **not reported** | | **204** | | **Apr 95** | | **2 y 2 m** | | **international** | | **26** | **3.6** | **not reported** | | **not reported** | **public** | **investigator** | |
| 7 Hascoet2005  RCT without poor recruitment | 814 | 936 | | 860 | | Jul 99 | | 1 y 7 m | | international | | 10 | 54.3 | not reported | | not reported | public and industry | investigator | |
| 7 Su and Chen2008  RCT without poor recruitment | 64 | not reported | | 65 | | Jul 00 | | 6 y | | national | | 1 | 10.8 | not reported | | not reported | not reported | investigator | |
| 7 Ballard2006  RCT without poor recruitment | 544 | 5129 | | 587 | | May 00 | | 4 y 11 m | | national | | 21 | 5.7 | not reported | | not reported | public | investigator | |
| **8 Grines2002**  **RCT with poor recruitment** | **430** | **not reported** | | **138** | | **not reported** | | **3y 3 m** | | **international** | | **12** | **3.5** | **not reported** | | **not reported** | **industry** | **probably investigator** | |
| 8 Grines 1993  RCT without poor recruitment | 370 | not reported | | 395 | | Jun 90 | | 1 y 10 m | | international | | not reported | not estimable | not reported | | not reported | not reported | probably investigator | |
| 8 Le May2001  RCT without poor recruitment | 124 | not reported | | 123 | | Aug 97 | | 1 y 10 m | | national | | 1 | 67.1 | not reported | | not reported | partly industry | probably investigator | |
| **8 Bonnefoy2002**  **RCT with poor recruitment** | **1200** | **not reported** | | **840** | | **Jun 97** | | **3 y 3m** | | **national** | | **27** | **9.6** | **not reported** | | **not reported** | **public and industry** | **probably investigator** | |
| 8 Schömig2000  RCT without poor recruitment | 140 | not reported | | 140 | | Dec 97 | | 1 y 2 m (assuming 6 months follow up time) | | national | | not reported | not estimable | not reported | | not reported | public and industry | probably investigator | |
| **8 Aversano2002**  **RCT with poor recruitment** | **2550** | **548** | | **451** | | **Jul 96** | | **2 y 11 m** | | **national** | | **11** | **14.1** | **not reported** | | **not reported** | **partly industry** | **probably investigator** | |
| **9 Höffken2007**  **RCT with poor recruitment** | **566** | **not reported** | | **161** | | **May 00** | | **1 y 9 m** | | **international** | | **39** | **2.4** | **not reported** | | **not reported** | **industry** | **industry** | |
| 9 Anzueto, 2006  RCT without poor recruitment | 400 | 432 | | 401 | | Nov 02 | | 1 y 5 m | | national | | 47 | 6.0 | not reported | | not reported | industry | industry | |
| **9 Ott, 2008**  **RCT with poor recruitment** | **154** | **not reported** | | **139** | | **Feb 01** | | **3 y 11 m** | | **national** | | **15** | **2.4** | **not reported** | | **not reported** | **industry** | **industry** | |
| 9 Portier, 2005  RCT without poor recruitment | 348 | not reported | | 349 | | Feb 01 | | 1 y 10 m | | national | | 80 | 2.4 | not reported | | not reported | industry | industry | |
| 9 Torres, 2008  RCT without poor recruitment | 608 | not reported | | 733 | | Jan 04 | | 1 y 5 m | | international | | 69 | 7.5 | not reported | | not reported | industry | industry | |
| 9 Welte, 2005  RCT without poor recruitment | 278 | not reported | | 397 | | Dec 01 | | 1 y 2 m | | international | | 54 | not estimable | not reported | | not reported | public and industry | industry | |
| **10 Malmström2012**  **RCT with poor recruitment** | **480** | **not reported** | | **342** | | **Feb 00** | | **9 y 4 m** | | **international** | | **28** | **1.3** | **not reported** | | **not reported** | **public and industry** | **investigator** | |
| 10 Brada, 2010  RCT without poor recruitment | 500 | not reported | | 447 | | Jun 03 | | 4 y 7 m | | national | | 36 | 2.7 | not reported | | not reported | public | investigator | |
| 10 Stupp, 2005  RCT without poor recruitment | 382 occured deaths | not reported | | 573 | | Aug 00 | | 1 y 6 m | | international | | 85 | 4.5 | not reported | | not reported | public and industry | investigator | |
| 10 Wick, 2012  RCT without poor recruitment | 412 | 584 | | 412 | | May 05 | | 4y 6 m | | international | | 24 | 3.8 | not reported | | not reported | industry | investigator | |
| **11 Pajk2008**  **RCT with poor recruitment** | **72** | **not reported** | | **47** | | **Sep 02** | | **2 y 3 m** | | **international** | | **8** | **2.6** | **not reported** | | **not reported** | **public and industry** | **industry** | |
| **11 Bachelot, 2011**  **RCT with poor recruitment** | **106 after amendment** | **not reported** | | **68** | | **Apr 04** | | **2 y 9 m** | | **national** | | **15** | **1.6** | **not reported** | | **not reported** | **industry** | **industry** | |
| 11 O`Shaughnessy JA, 2001  RCT without poor recruitment | 85 | not reported | | 95 | | May 96 | | 1 y 0 m | | international | | 23 | 24.8 | not reported | | not reported | industry | industry | |
| 11 O`Shaughnessy J, 2002  RCT without poor recruitment | 500 | not reported | | 511 | | not reported | | not reported | | international | | 75 | #DIV/0! | not reported | | not reported | industry | industry | |
| 11 Mavroudis, 2010  RCT without poor recruitment | 272 | 307 | | 286 | | May 02 | | 5 y 6 m | | national | | not reported | not estimable | not reported | | not reported | not reported | not reported | |
| **11 Stockler, 2011**  **RCT with poor recruitment** | **465** | **not reported** | | **325** | | **July 01** | | **3 y 11 m** | | **not reported** | | **34** | **2.4** | **not reported** | | **not reported** | **industry** | **industry** | |
| 11 Stemmler, 2011  RCT without poor recruitment | 141 | not reported | | 141 | | 2003 (month not reported) | | 3 y | | national | | 24 | 2.0 | not reported | | not reported | industry | industry | |
| **11 Talbot, 2002**  **RCT with poor recruitment** | **76** | **not reported** | | **42** | | **May 96** | | **0 y 10 m** | | **international** | | **18** | **2.8** | **not reported** | | **not reported** | **industry** | **industry** | |
| 11 Wardley, 2010  RCT without poor recruitment | 220 | not reported | | 225 | | Feb 02 | | 3y 7 m | |  | | 43 | 1.5 | not reported | | not reported | industry | industry | |
| **12 Perry2012**  **RCT with poor recruitment** | **512** | **563** | | **186** | | **Oct 02** | | **3 y 7 m** | | **international** | | **15** | **3.5** | **not reported** | | **not reported** | **industry** | **probably investigator** | |
| **12 Sideras2006**  **RCT with poor recruitment** | **530** | **not reported** | | **141** | | **Dec 98** | | **not reported** | | **national** | | **not reported** | **#DIV/0!** | **not reported** | | **not reported** | **partly public** | **investigator** | |
| 12 Kakkar2004  RCT without poor recruitment | 366 | not reported | | 385 | | May 95 | | 5 y 11 m | | international | | 10 | 6.5 | not reported | | not reported | industry | industry | |
| 12 Agnelli2009  RCT without poor recruitment | 1080 | not reported | | 1166 | | Oct 03 | | 3 y 7 m | | national | | 62 | 5.2 | not reported | | not reported | industry | industry | |
| **13 Sandercock2012**  **RCT with poor recruitment** | **6000** | **not reported** | | **3035** | | **May 00** | | **11 y 2 m** | | **international** | | **156** | **1.7** | **names given but not their profession** | | **not reported** | **public** | **investigator** | |
| 13 Hacke 1995  RCT without poor recruitment | 600 | not reported | | 620 | | 1992 (months not reported) | | 1 y 2 m (see discussion) | | international | | 75 | 7.1 | not reported | | not reported | industry | industry | |
| 13 Hacke 1998  RCT without poor recruitment | 800 | 814 | | 800 | | Oct 96 | | 1 y 3 m | | international | | 108 | 5.9 | not reported | | not reported | not reported | probably industry | |
| 13 Hacke 2008  RCT without poor recruitment | 800 | not reported | | 821 | | Jul 03 | | 4 y 4 m | | international | | 130 | 1.5 | not reported | | not reported | probably industry | industry | |
| 13 no authors listed ("…stroke study group"); NEJM 1995; part 2  RCT without poor recruitment | Part 1: 280; Part 2 320 | not reported | | 624 | | Jan 91 | | 3 y 9 m | | national | | not reported | not estimable | not reported | | not reported | partly public | probably investigator | |
| 13 Davis 2008  RCT without poor recruitment | 100 | 3908 | | 101 | | Apr 01 | | 5 y 9 m | | international | | 15 | 1.2 | not reported | | not reported | public | investigator | |
| **14 Smith2007**  **RCT with poor recruitment** | **600** | **not reported** | | **158** | | **May 01** | | **3 y 1 m** | | **national** | | **14** | **3.7** | **not reported** | | **not reported** | **public** | **investigator** | |
| 14 Bisits2004  RCT without poor recruitment | 240 | 1174 | | 238 | | Apr 97 | | 3 y 1 m | | international | | 4 | 19.3 | not reported | | not reported | public | investigator | |
| **15 Wenzel2004**  **RCT with poor recruitment** | **1500** | **5967** | | **1219** | | **Jun 99** | | **2 y 9 m** | | **international** | | **44** | **10.1** | **not reported** | | **not reported** | **public and industry** | **investigator** | |
| 15 Callaway 2006  RCT without poor recruitment | 324 | 701 | | 325 | | May 03 | | 1y 11m | | national | | 1 | 300.0 | not reported | | contact to community groups, public disclosure by newspaper, television, public forums, email site and call in telephone number | public | investigator | |
| 15 Mentzelopoulos 2009  RCT without poor recruitment | 68 | 139 | | 100 | | Jun 06 | | 9 m | | national | | 1 | 133.3 | not reported | | not reported | public | investigator | |
| 15 Lidner 1997  RCT without poor recruitment | 38 | not reported | | 40 | | Jul 94 | | 1 y 5 m | | national | | 1 | 68.6 | not reported | | not reported | public | investigator | |
| 15 Gueugniaud 2008  RCT without poor recruitment | 2416 | not reported | | 2956 | | May 04 | | 1 y 11m | | national | | 31 | 49.8 | not reported | | not reported | public | investigator | |
| 15 Stiell 2001  RCT without poor recruitment | 200 | 324 | | 200 | | Jul 97 | | 1 y 4 m | | international | | 3 | 50.0 | not reported | | not reported | public | investigator | |

*rough estimate for recruitment speed based on own calculations (number of patients recruited devided by recruitment duration in years and number of study centers); not adjusted to the time a site was actually open for recruitment, because this was not reported in the publications of the included trials.

**based on duration of hospital stay; duration of study conduct was 2 y; follow-up duration for the survival analysis not reported
