# Supplementary material for: Comparison of randomized controlled trials discontinued or revised for poor recruitment and completed trials with the same research question: a matched qualitative study
Source: Trials. 2019 Dec 30;20:800. doi: 10.1186/s13063-019-3957-4 (PMC6937940; doi:10.1186/s13063-019-3957-4)
Supplement: Supplementary file 6 — Additional file 6. General characteristics of included randomized controlled trials (RCTs) [file 13063_2019_3957_MOESM6_ESM.docx]

**APPENDIX F: General characteristics of included randomized controlled trials (RCTs)**

| **Included RCTs** | **Country** | **Patients randomized, n** | **Disease /patient population** | **Comparison** | **Primary outcome** |
| --- | --- | --- | --- | --- | --- |
| **1 Anvari2011**  **RCT with poor recruitment** | Canada | 104 | gastroesophageal reflux disease | laparoscopic Nissen fundoplication vs. proton pumps inhibitors | GERD symptoms |
| 1 Galmiche2011  RCT without poor recruitment | Europe (no details on countries) | 554 | gastroesophageal reflux disease | laparoscopic antireflux surgery vs. esomeprazole | time to treatment failure |
| **1 Grant2008**  **RCT with poor recruitment** | United Kingdom | 357 | gastroesophageal reflux disease | laparoscopic fundoplication vs. Proton pumps inhibitors | GERD symptoms |
| 1 Mahon2004  RCT without poor recruitment | United Kingdom | 217 | gastroesophageal reflux disease | laparoscopic Nissen fundoplication vs. proton pumps inhibitors | not reported |
| **2 Bonneterre2004**  **RCT with poor recruitment** | France | 142 | metastatic breast cancer | epirubicin plus docetaxel vs. epirubicin plus fluorouacil and cyclophosphamide | objective tumor response rate |
| 2 Nabholtz2003  RCT without poor recruitment | Europe, South Africa, South America (no details on countries), Australia, and Canada | 429 | metastatic breast cancer | doxorubicin plus docetaxel vs. doxorubicin plus cyclophosphamide | time to progression |
| **2 Bontenbal2005**  **RCT with poor recruitment** | The Netherlands | 216 | metastatic breast cancer | doxorubicin plus docetaxel vs. doxorubicin plus cyclophosphamide and fluoracil | time to progression |
| **2 Blohmer2010**  **RCT with poor recruitment** | Germany | 240 | metastatic breast cancer | epirubicin plus cyclophosphamide vs. epirubicin plus docetaxel | objective overall response rate |
| 2 Biganzoli2002  RCT without poor recruitment | not reported | 275 | metastatic breast cancer | doxorubicin plus paclitaxel vs. doxorubicin plus cyclophosphamide | progression-free survival |
| 2 Jassem2001  RCT without poor recruitment | Central and Eastern Europe and Israel (Poland, Yugoslavia, Russia, Croatia, Latvia, Hungary, Israel, Belgium) | 267 | metastatic breast cancer | doxorubicin plus paclitaxel vs. doxorubicin plus cyclophosphamide and fluoracil | time to disease progression |
| **3 Campos2009**  **RCT with poor recruitment** | USA and Europe | 130 | advanced breast cancer in postmenopausal women | exemstane vs anastrozole | response rate |
| 3 Llombart-Cussac2012  RCT without poor recruitment | Spain | 103 | advanced breast cancer in postmenopausal women | examestane vs anastrozole | objective response rate |
| **4 Connolly2006**  **RCT with poor recruitment** | Canada, US, Germany, Austria, Sweden, England | 412 | cardiac arrhythmia | standard beta-blocker vs amiodarone+beta-blocker vs sotalole | first occurence of any shock from ICD |
| 4 Kowey2011  RCT without poor recruitment | 26 (including Argentina, Canada, USA, Australia, European countries, Japan, Mexico) | 486 | ventricular arrhythmia | placebo vs cevilarone 50mg vs cevilarone 100mg vs cevilarone 300mg | first occurence of shock from ICD or death |
| 4 Dorian2004  RCT without poor recruitment | USA and Europe (no details on countries) | 633 | ventricular arrythmia | placebo vs azimilide 75mg vs azimilide 125 mg | (1) all-cause shocks plus symptomatic tachyarrhythmias terminated by ATP and (2) all-cause shocks |
| **5 Dellinger2007**  **RCT with poor recruitment** | North America and Europe (no details on countries) | 100 | confirmed diagnosis of necrotising pancreatitis within 120 h of symptom onset | meropenem vs. placebo | pancreatic infection rate |
| **5 Rokke2007**  **RCT with poor recruitment** | Norway | 73 | acute pancreatitis | imipenem vs. usual care | infectious complications |
| **5 Garcia-Barrasa2009**  **RCT with poor recruitment** | Spain | 46 | acute necrotising pancreatitis | ciprofloxacin vs. placebo | infected pancreatic necrosis |
| **6 Doyle2006**  **RCT with poor recruitment** | Australia, Canada, New Zealand | 70 | neonates birthweight <1000g, requiring ventilator support | dexamethasone vs. placebo | survival free of major neurosensory disability;primary outcome was changed to failed extubation after 10 days |
| 6 Brozanski 1995  RCT without poor recruitment | USA | 88 | neonates birthweight ≤1500g, requiring ventilator support | dexamethasone vs. placebo | survival without supplemental oxygen |
| 6 Durand 1995  RCT without poor recruitment | USA | 44 | neonates birthweight 501-1500g, requiring ventilator support | dexamethasone vs. usual care | improvement in respiratory system complicance |
| 6 Cummings 1989  RCT without poor recruitment | USA | 36 | neonates birthweight 1250g, requiring ventilator support | dexamethasone vs. placebo | poor pulmonary outcome (dependence on mechanical ventilation at 60 days of age or death from respiratory failure) |
| **6 Kari 1993**  **RCT with poor recruitment** | Finland | 41 | neonates birthweight ≤1500g, requiring ventilator support | dexamethasone vs. placebo | poor pulmonary outcome (dependence on mechanical ventilation at 28 days of age or death from respiratory failure) |
| 6 Kovacs 1998  RCT without poor recruitment | Canada | 60 | neonates birthweight ≤1500g, requiring ventilator support | dexamethasone plus nebulized budesonide vs. placebo | chronic lung disease |
| 6 Ohlsson 1992  RCT without poor recruitment | Canada | 25 | neonates birthweight ≤1500g, requiring ventilator support | dexamethasone vs. sham | extubation within 7 days |
| 6 Walther 2003  RCT without poor recruitment | USA | 36 | neonates birthweight >600g (preterm) with repiratory distress syndrome requiring ventilator support | dexamethasone vs. placebo | serum cortisol and extubation within 7-14 d |
| 6 Kazzi1990  RCT without poor recruitment | USA | 23 | neonates birthweight <1500g with bronchopulmonary dysplasia requiring ventilator support | dexamethasone followed by hydrocortisone vs. placebo | extubation within 8 days of therapy |
| **7 Field2005**  **RCT with poor recruitment** | United Kingdom, Ireland, Belgium, Spain, Switzerland | 108 | infants of less than 34 weeks’ gestation, aged 28 days, and with severe respiratory failure requiring ventilatory support | ventilatory gas with nitric oxide vs ventilatory support without nitric oxide | death or severe disability at 1 year (as a composite outcome and separately), and death before discharge from hospital or chronic lung disease |
| 7 Kinsella 2006  RCT without poor recruitment | USA | 793 | gestational age of 34 weeks or less, birth within the previous 48 hours, respiratory failure requiring endotracheal intubation and mechanical ventilation, and a birth weight of 500 to 1250 g | ventilatory gas with nitric oxide vs ventilatory support without nitric oxide | death or bronchopulmonary dysplasia |
| 7 Schreiber2003  RCT without poor recruitment | USA | 207 | gestational age of 34 weeks or less, birth within the previous 72 hours, respiratory failure requiring endotracheal intubation and mechanical ventilation, and a birth weight of less than 2000 g | ventilatory gas with nitric oxide vs ventilatory support without nitric oxide | death or chronic lung disease (among surviving infants). |
| **7 Trial Group 1999***  **RCT with poor recruitment** | France, Belgium | 204 | preterm or near term infants with neonatal respiratory failure | ventilatory gas with nitric oxide vs ventilatory support without nitric oxide | oxygenation index at 2 h |
| 7 Hascoet2005  RCT without poor recruitment | France, Belgium | 860 | gestational age <32 weeks gestation at birth --> 15% expected with hypoxemic respiratory failure | ventilatory gas with nitric oxide vs ventilatory support without nitric oxide | intact survival at 28 days of age |
| 7 Su and Chen2008  RCT without poor recruitment | Taiwan | 65 | critically ill preterm neonates who who required mechanical ventilation | ventilatory gas with nitric oxide vs ventilatory support without nitric oxide | mean oxygenation index 24 h after randomization |
| 7 Ballard2006  RCT without poor recruitment | USA | 587 | infants with a birth weight of 1250 g or less who required ventilatory support between 7 and 21 days of age | ventilatory gas with nitric oxide vs ventilatory support without nitric oxide | survival withou bronchopulmonary dysplasia at 36 weeks |
| **8 Grines2002**  **RCT with poor recruitment** | USA, Finland, Argentina | 138 | acute myocardial infarction <12 h | transfer for primary angioplasty vs. onsite thrombolytic therapy | death, non-fatal reinfarction or disabling stroke at 30 days |
| 8 Grines 1993  RCT without poor recruitment | USA, France | 395 | acute myocardial infarction <12 h | primary angioplasty vs. onsite thrombolytic therapy | death or recurrent ischemia |
| 8 Le May2001  RCT without poor recruitment | Canada | 123 | acute myocardial infarction <12 h | primary angioplasty vs. onsite thrombolytic therapy | death or reinfarction or stroke or repeat target vessel revascularization for ischemia at 6 months |
| **8 Bonnefoy2002**  **RCT with poor recruitment** | France | 840 | acute myocardial infarction <6 h | primary angioplasty vs. onsite thrombolytic therapy | death, non-fatal reinfarction or disabling stroke at 30 days |
| 8 Schömig2000  RCT without poor recruitment | Germany | 140 | acute myocardial infarction <12 h | primary angioplasty vs. onsite thrombolytic therapy | salvage index (scintigraphy) |
| **8 Aversano2002**  **RCT with poor recruitment** | USA | 451 | acute myocardial infarction <12 h | primary angioplasty vs. onsite thrombolytic therapy | death, reinfarction or stroke within six months |
| **9 Höffken2007**  **RCT with poor recruitment** | Austria, Germany, Switzerland, Turkey, Greece, Lithuania, Mexico, Finland, Poland, Spain, Australia, Israel, Hungary, Canada, | 161 | hospital-acquired pneumonia | moxifloxacin (first i.v. then oral) vs. ceftriaxone (i.v.) then cefuroxime axetil (oral) | clinical response 7–10 days after the end of a 7–14-day treatment period |
| 9 Anzueto, 2006  RCT without poor recruitment | USA | 401 | community acquired pneumonia in the elderly | moxifloxacin vs levofloxacin | clinical response at the test-of-cure visit (between days 5 and 21 after the end of therapy) |
| **9 Ott, 2008**  **RCT with poor recruitment** | Germany | 139 | hospital aquired pneumonia (aspiration pneumonia or lung abscess) | moxifloxacin vs ampicillin/sulbactam | clinical response determined at the test of cure visit 5-14 days after last treatment |
| 9 Portier, 2005  RCT without poor recruitment | France | 349 | community acquired pneumonia patients with risk factors | moxifloxacin vs amoxicillin-clavulanate plus roxithromycin | clinical response, defined as clinical success or nonsuccess at the test of cure visit 5-7 days post treatment |
| 9 Torres, 2008  RCT without poor recruitment | 17 Countries (Europe, Latin America and South Africa) | 733 | community-acquired pneumonia in hospitalised patients | moxifloxacin vs ceftriaxone plus levofloxacin | clinical response at test of cure (4–14 days after the completion of treatment). |
| 9 Welte, 2005  RCT without poor recruitment | Germany, France, Lithuania, Poland | 397 | community-acquired  pneumonia in patients who received initial parenteral therapy | moxifloxacin vs ceftriaxone with or without erythromycin | clinical response at the test-of-cure visit 5–20 days after receipt of the final dose of the study drug |
| **10 Malmström2012**  **RCT with poor recruitment** | Austria, Denmark, Norway, Sweden, Switzerland, Turkey | 342 | patients with glioblastoma who are older than 60 years | temozolomide vs hyperfractionated radiotherapy vs standard radiotherapy | overall survival |
| 10 Brada, 2010  RCT without poor recruitment | United Kingdom | 447 | adults with progressive glioblastoma and prior radiotherapy | temozolomide vs procarbazine, lomustine, and vincristine (PCV) | survival and 12 week progression free survival |
| 10 Stupp, 2005  RCT without poor recruitment | Belgium, Germany, Spain, France, Netherlands, Italy, Poland, Israel, Sweden, Slovenia, UK, Austria, Canada, Switzerland | 573 | adults with newly diagnosed and histologically confirmed glioblastoma | temozolomide plus radiotherapy vs radiotherapy alone | overall survival |
| 10 Wick, 2012  RCT without poor recruitment | Germany and Switzerland | 412 | patients with anaplastic astrocytoma or glioblastoma >65 years | temozolomide vs standard radiotherapy | overall survival |
| **11 Pajk2008**  **RCT with poor recruitment** | not reported | 47 | metastatic breast cancer pretreated with anthracyclines and taxanes | oral capecitabine vs i.v. vinorelbine | response rate |
| **11 Bachelot, 2011**  **RCT with poor recruitment** | France | 68 | metastatic breast cancer | oral capecitabine plus i.v. docetaxel vs. i.v. epirubicin plus docetaxel | non-progression rate 6 months after randomization |
| 11 O`Shaughnessy, 2001  RCT without poor recruitment | USA, Canada, Europe, and Australia | 95 | advanced/metastatic breast cancer; at least 55years old | oral capecitabine vs i.v. cyclophosphamide, methotrexate, 5-fluorouracil (CMF) | response rate |
| 11 O`Shaughnessy, 2002  RCT without poor recruitment | Argentina, Australia, Brazil, Canada, France, Germany, Israel, Italy, Mexico, New Zealand, Norway, Russia, Spain, Taiwan, the United Kingdom, and USA | 511 | advanced/metastatic breast cancer; at least 55years old | oral capecitabine plus i.v. docetaxel vs i.v. docetaxel | time to disease progression |
| 11 Mavroudis, 2010  RCT without poor recruitment | Greece | 286 | advanced/metastatic breast cancer; at least 55years old | oral capecitabine plus i.v. docetaxel vs. i.v. epirubicin plus i.v. docetaxel | time to disease progression |
| **11 Stockler, 2011**  **RCT with poor recruitment** | not reported | 325 | advanced breast cancer | oral capecitabine (administered intermittently or continuously) vs classical oral cyclophosphamide, i.v. methotrexate, and fluorouracil (CMF) | quality-adjusted progression-free survival |
| 11 Stemmler, 2011  RCT without poor recruitment | Germany | 141 | metastatic breast cancer | oral capecitabine plus i.v. gemcitabine vs vinorelbine plus gemcitabine vs i.v. cisplatin plus gemcitabine | response rate |
| **11 Talbot, 2002**  **RCT with poor recruitment** | Australia, Belgium, Canada, France, Poland, Russia, Spain, UK, USA | 42 | advanced and/or metastatic breast cancer | oral continuous capecitabine vs i.v. paclitaxel | overall response rate |
| 11 Wardley, 2010  RCT without poor recruitment | not reported | 225 | metastatic breast cancer | oral capecitabine plusi.v. trastuzumab plus i.v. docetaxel vs i.v. trastuzumab plus docetaxel | overall response rate |
| **12 Perry2010**  **RCT with poor recruitment** | USA, Canada, Australia, Italy | 186 | glioma | heparin vs. placebo | objectively documented symptomatic deep vein thrombosis or pulmonary embolism at 6 months |
| **12 Sideras2006**  **RCT with poor recruitment** | USA | 141 | different advanced cancers e.g. advanced breast cancer | heparin vs. no intervention | overall survival |
| 12 Kakkar2004  RCT without poor recruitment | UK, Canada, Italy | 385 | different advanced cancers e.g. advanced breast cancer | heparin vs. placebo | overall survival at 1 year |
| 12 Agnelli2009  RCT without poor recruitment | Italy | 1166 | different types of metastatic or locally advanced cancers | heparin vs. placebo | symptomatic deep vein thrombosis or pulmonary embolism or visceral or cerebral venous thrombosis, acute myocardial infarction, ischeamic stroke, acute peripheral arterial thromboembolism, unexplained death of possible thromboembolic origin during study period plus 10 days |
| **13 Sandercock2012**  **RCT with poor recruitment** | UK, Austria, Belgium, Switzerland, Norway, Sweden, Australasia, Italy, Portugal, Poland, Canada, Mexico | 3035 | acute ischaemic stroke | thrombolytic therapy vs. control (standard of care) | patients alive and independent as measured by the Oxford handicap score at 6 month |
| 13 Hacke 1995  RCT without poor recruitment | Germany, Austria, Switzerland, Italy, finland, Norway, Sweden, Denmark, UK, Netherlands, belgium, France, Portugal, Spain | 620 | acute ischaemic stroke | thrombolytic therapy vs. placebo | activities of daily living (Barthel Index) and modified Rankin Scale at day 90 |
| 13 Hacke 1998  RCT without poor recruitment | Australia, Austria, Belgium, Denmark, Finland,France, Germany, Italy, Netherlands, Norway, Portugal, Spain, Sweden, Switzerland, UK | 800 | acute ischaemic stroke | thrombolytic therapy vs. placebo | patients who had favourable outcome on the modified Rankin scale at day 90 |
| 13 Hacke 2008  RCT without poor recruitment | Austria, Belgium, Czech Republic, Denmark, Finland, France, Germany, Greece, Hungary, Italy, Netherlands, Poland, Portugal, Slovakia, Spain, Sweden, Switzerland, United Kingdom | 821 | acute ischaemic stroke | thrombolytic therapy vs. placebo | disability at day 90 (modified Rankin scale) |
| 13 Stroke study group 1995  RCT without poor recruitment | USA | 624 | acute ischaemic stroke | thrombolytic therapy vs. placebo | four primary disability outcomes (global test, Barthel index, modified Rankin scale, Glasgow outcome scale, NIHSS |
| 13 Davis 2008  RCT without poor recruitment | Australia, New Zealand, Belgium, UK | 101 | acute hemispheric ischaemic stroke | thrombolytic therapy vs. placebo | geometric mean of infarct growth until day 90 |
| **14 Smith2007**  **RCT with poor recruitment** | Canada | 158 | laboring women between 24 and 32 weeks | nitroglycerin vs. placebo | composite outcome of neonatal morbidity associated with long term morbidity and perinatal mortality |
| 14 Bisits2004  RCT without poor recruitment | Singapore, Hongkong, Australia | 238 | laboring women between 24 and 35 weeks (preterm delivery risk) | nitroglycerin vs. usual care (i.v. beta 2 sympathomimetic) | number of days from randomization to delivery |
| **15 Wenzel2004**  **RCT with poor recruitment** | Austria, Germany and Switzerland | 1219 | patients with an out-of-hospital cardiac arrest and ventricular fibrillation | epinephrine vs. vasopressin | survival until hospital admission |
| 15 Callaway 2006  RCT without poor recruitment | USA | 325 | patients with an out-of-hospital cardiac arrest | vasopressin plus epinephrine vs. placebo and epinephrine | survival until hospital admission (restoration of spontaneous circulation at any time during resuscitation and the presence of pulses at hospital arrival) |
| 15 Mentzelopoulos 2009  RCT without poor recruitment | Greece | 100 | patients with an in-hospital cardiac arrest and ventricular fibrillation | vasopressin plus epinephrine vs. placebo and epinephrine | acccording to sample size calculation: survival to hopsital discharge, other primary outcomes reported: return of spontaneous circulation for 15 minutes or longer |
| 15 Lidner 1997  RCT without poor recruitment | Germany | 40 | patients with an out-of-hospital cardiac arrest and ventricular fibrillation | vasopressin vs. epinephrine | survival until hospital admission |
| 15 Gueugniaud 2008  RCT without poor recruitment | France | 2956 | patients with an out-of-hospital cardiac arrest and ventricular fibrillation | vasopressin plus epinephrine vs placebo plus epinephrine | survival until hospital admission |
| 15 Stiell 2001  RCT without poor recruitment | Canada, UK | 200 | patients with an in-hospital cardiac arrest , cardiac fibrillation only in some patients | vasopressin vs. epinephrine | acccording to sample size calculation: survival at 1h, other primary outcomes reported: survival to hospital discharge,  and neurological function |
