# Supplementary material for: Comparison of randomized controlled trials discontinued or revised for poor recruitment and completed trials with the same research question: a matched qualitative study
Source: Trials. 2019 Dec 30;20:800. doi: 10.1186/s13063-019-3957-4 (PMC6937940; doi:10.1186/s13063-019-3957-4)
Supplement: Supplementary file 7 — Additional file 7. Reporting quality of the recruitment process in included randomized controlled trials [file 13063_2019_3957_MOESM7_ESM.docx]

**APPENDIX G: Reporting quality of the recruitment process in included randomized controlled trials**

| **Included randomized controlled trials** | **Was the anticipated recruitment duration reported?** | **Was the anticipated prevalence of eligible patients reported?** | **Was there a detailed and comprehensive patient flow (including number of patients screened for eligibility, not eligible, not giving consent, and randomized)?** | **Was the location/ care facility where patients were recruited reported?** | **Was reported by whom patients were recruited?** | **Was the actual recruitment period or duration reported?** |
| --- | --- | --- | --- | --- | --- | --- |
| **1 Anvari et al. 2011**  **RCT with poor recruitment** | **No** | **No** | **Yes** | **Yes** | **No** | **Yes** |
| 1 Galmiche et al. 2011  RCT without poor recruitment | No | No | Yes | No | No | No |
| **1 Grant** **et al. 2008**  **RCT with poor recruitment** | **No** | **No** | **Yes** | **Yes** | **Yes** | **Yes** |
| 1 Mahon et al. 2004  RCT without poor recruitment | No | No | No | No | Yes | No |
| **2 Bonneterre** **et al. 2004**  **RCT with poor recruitment** | **No** | **No** | **No** | **No** | **No** | **Yes** |
| 2 Nabholtz et al. 2003  RCT without poor recruitment | No | No | No | No | No | No |
| **2 Bontenbal** **et al. 2005**  **RCT with poor recruitment** | **No** | **No** | **No** | **Yes** | **No** | **Yes** |
| **2 Blohmer** **et al. 2010**  **RCT with poor recruitment** | **No** | **No** | **No** | **No** | **No** | **Yes** |
| 2 Biganzoli et al. 2002  RCT without poor recruitment | No | No | No | No | No | Yes |
| 2 Jassem et al. 2001  RCT without poor recruitment | No | No | No | No | No | Yes |
| **3 Campos** **et al. 2009**  **RCT with poor recruitment** | **No** | **No** | **No** | **No** | **No** | **Yes** |
| 3 Llombart-Cussac et al. 2012  RCT without poor recruitment | No | No | No | No | No | Yes |
| **4 Connolly** **et al. 2006**  **RCT with poor recruitment** | **Yes** | **Yes** | **No** | **Yes** | **No** | **Yes** |
| 4 Kowey et al. 2011  RCT without poor recruitment | No | No | No | Yes | No | Yes |
| 4 Dorian et al. 2004  RCT without poor recruitment | No | No | No | No | No | Yes |
| **5 Dellinger** **et al. 2007**  **RCT with poor recruitment** | **No** | **No** | **Yes** | **No** | **No** | **Yes** |
| **5 Rokke** **et al. 2007**  **RCT with poor recruitment** | **No** | **No** | **No** | **Yes** | **No** | **Yes** |
| **5 Garcia-Barrasa** **et al. 2009**  **RCT with poor recruitment** | **No** | **No** | **Yes** | **Yes** | **No** | **Yes** |
| **6 Doyle** **et al. 2006**  **RCT with poor recruitment** | **No** | **No** | **No** | **No** | **No** | **Yes** |
| 6 Brozanski et al. 1995  RCT without poor recruitment | No | No | No | Yes | No | Yes |
| 6 Durand et al. 1995  RCT without poor recruitment | No | No | No | Yes | No | No |
| 6 Cummings et al. 1989  RCT without poor recruitment | No | No | No | Yes | No | Yes |
| **6 Kari et al. 1993**  **RCT with poor recruitment** | **No** | **No** | **No** | **Yes** | **No** | **Yes** |
| 6 Kovacs et al. 1998  RCT without poor recruitment | No | No | Yes | Yes | No | Yes |
| 6 Ohlsson et al. 1992  RCT without poor recruitment | No | No | No | Yes (based on author affiliation) | No | Yes |
| 6 Walther et al. 2003  RCT without poor recruitment | No | No | No | Yes | No | Unclear, only period of study conduct reported |
| 6 Kazzi et al. 1990  RCT without poor recruitment | No | No | No | Yes | No | Yes |
| **7 Field** **et al. 2005**  **RCT with poor recruitment** | **No** | **No** | **No** | **Yes** | **No** | **Yes** |
| 7 Kinsella et al. 2006  RCT without poor recruitment | No | No | Yes | Yes | No | Yes |
| 7 Schreiber et al. 2003  RCT without poor recruitment | No | No | No | Yes | No | Yes |
| **7 Trial Group 1999^a^**  **RCT with poor recruitment** | **No** | **No** | **No** | **Yes** | **No** | **Yes** |
| 7 Hascoet et al. 2005  RCT without poor recruitment | No | No | Yes | Yes | No | Yes |
| 7 Su and Chen2008  RCT without poor recruitment | No | No | No | Yes | No | Yes |
| 7 Ballard et al. 2006  RCT without poor recruitment | No | No | Yes | Yes | No | Yes |
| **8 Grines** **et al. 2002**  **RCT with poor recruitment** | **No** | **No** | **No** | **Yes** | **No** | **Yes** |
| 8 Grines et al. 1993  RCT without poor recruitment | No | No | No | Yes | No | Yes |
| 8 Le May et al. 2001  RCT without poor recruitment | No | No | No | Yes | No | Yes |
| **8 Bonnefoy** **et al. 2002**  **RCT with poor recruitment** | **No** | **No** | **No** | **Yes** | **No** | **Yes** |
| 8 Schömig et al. 2000  RCT without poor recruitment | No | No | No | No | No | Yes |
| **8 Aversano** **et al. 2002**  **RCT with poor recruitment** | **No** | **No** | **Yes** | **Yes** | **No** | **Yes** |
| **9 Höffken** **et al. 2007**  **RCT with poor recruitment** | **No** | **No** | **No** | **No** | **No** | **Yes** |
| 9 Anzueto et al. 2006  RCT without poor recruitment | No | No | Yes | No | No | Yes |
| **9 Ott et al. 2008**  **RCT with poor recruitment** | **No** | **No** | **No** | **No** | **No** | **Yes** |
| 9 Portier et al. 2005  RCT without poor recruitment | No | No | No | No | No | Yes |
| 9 Torres et al. 2008  RCT without poor recruitment | No | No | No | No | No | Yes |
| 9 Welte et al. 2005  RCT without poor recruitment | No | No | No | No | No | Yes |
| **10 Malmström** **et al. 2012**  **RCT with poor recruitment** | **No** | **No** | **No** | **Yes** | **No** | **Yes** |
| 10 Brada et al. 2010  RCT without poor recruitment | Yes | Yes | No | No | No | Yes |
| 10 Stupp et al. 2005  RCT without poor recruitment | No | No | No | No | No | Yes |
| 10 Wick et al. 2012  RCT without poor recruitment | Yes | Yes | Yes | No | No | Yes |
| **11 Pajk** **et al. 2008**  **RCT with poor recruitment** | **No** | **No** | **No** | **No** | **No** | **Yes** |
| **11 Bachelot et al. 2011**  **RCT with poor recruitment** | **No** | **No** | **No** | **No** | **No** | **Yes** |
| 11 O`Shaughnessy JA et al. 2001  RCT without poor recruitment | No | No | No | No | No | Yes |
| 11 O`Shaughnessy J, et al. 2002  RCT without poor recruitment | No | No | No | No | No | No |
| 11 Mavroudis et al. 2010  RCT without poor recruitment | Yes | Yes | Yes | No | No | Yes |
| **11 Stockler et al. 2011**  **RCT with poor recruitment** | **Yes** | **Yes** | **No** | **No** | **No** | **Yes** |
| 11 Stemmler et al. 2011  RCT without poor recruitment | No | No | No | No | No | Yes |
| **11 Talbot et al. 2002**  **RCT with poor recruitment** | **No** | **No** | **No** | **No** | **No** | **Yes** |
| 11 Wardley et al. 2010  RCT without poor recruitment | No | No | No | No | No | Yes |
| **12 Perry** **et al. 2012**  **RCT with poor recruitment** | **No** | **No** | **Yes** | **Yes** | **No** | **Yes** |
| **12 Sideras** **et al. 2006**  **RCT with poor recruitment** | **No** | **No** | **No** | **Yes** | **No** | **No (only study conduct period)** |
| 12 Kakkar et al. 2004  RCT without poor recruitment | No | No | No | Yes | No | Yes |
| 12 Agnelli2 et al. 009  RCT without poor recruitment | No | No | No | No | No | Yes |
| **13 Sandercock** **et al. 2012**  **RCT with poor recruitment** | **No** | **No** | **No** | **Yes** | **Yes** | **Yes** |
| 13 Hacke et al. 1995  RCT without poor recruitment | No | No | No | No | No | Yes |
| 13 Hacke et al. 1998  RCT without poor recruitment | No | No | Yes | No | No | Yes |
| 13 Hacke et al. 2008  RCT without poor recruitment | No | No | No | No | No | Yes |
| 13 No authors listed ("…stroke study group"); NEJM 1995; part 2  RCT without poor recruitment | No | No | No | No | No | Yes |
| 13 Davis et al. 2008  RCT without poor recruitment | No | No | Yes | No | No | Yes |
| **14 Smith** **et al. 2007**  **RCT with poor recruitment** | **No** | **No** | **No** | **Yes** | **No** | **Yes** |
| 14 Bisits et al. 2004  RCT without poor recruitment | No | No | Yes | Yes | No | Yes |
| **15 Wenze** **et al. l2004**  **RCT with poor recruitment** | **No** | **No** | **Yes** | **Yes** | **No** | **Yes** |
| 15 Callaway et al. 2006  RCT without poor recruitment | No | No | Yes | Yes | No | Yes |
| 15 Mentzelopoulos et al. 2009  RCT without poor recruitment | No | No | Yes | Yes | No | Yes |
| 15 Lidner et al. 1997  RCT without poor recruitment | No | No | No | Yes | No | No |
| 15 Gueugniaud et al. 2008  RCT without poor recruitment | No | No | No | Yes | No | Yes |
| 15 Stiell et al. 2001  RCT without poor recruitment | No | No | Yes | Yes | No | Yes |

*RCT* randomized controlled trial

^a^The Franco-Belgium Collaborative Nitric Oxide Trial Group
